# A unified view on weakly correlated recurrent networks

**DOI:** 10.3389/fncom.2013.00131

**Published:** 2013-10-18

**Authors:** Dmytro Grytskyy, Tom Tetzlaff, Markus Diesmann, Moritz Helias

**Affiliations:** ^1^Institute of Neuroscience and Medicine (INM-6) and Institute for Advanced Simulation (IAS-6), Jülich Research Centre and JARAJülich, Germany; ^2^Medical Faculty, RWTH Aachen UniversityGermany

**Keywords:** correlations, linear response, Hawkes process, leaky integrate-and-fire model, binary neuron, linear rate model, Ornstein–Uhlenbeck process

## Abstract

The diversity of neuron models used in contemporary theoretical neuroscience to investigate specific properties of covariances in the spiking activity raises the question how these models relate to each other. In particular it is hard to distinguish between generic properties of covariances and peculiarities due to the abstracted model. Here we present a unified view on pairwise covariances in recurrent networks in the irregular regime. We consider the binary neuron model, the leaky integrate-and-fire (LIF) model, and the Hawkes process. We show that linear approximation maps each of these models to either of two classes of linear rate models (LRM), including the Ornstein–Uhlenbeck process (OUP) as a special case. The distinction between both classes is the location of additive noise in the rate dynamics, which is located on the output side for spiking models and on the input side for the binary model. Both classes allow closed form solutions for the covariance. For output noise it separates into an echo term and a term due to correlated input. The unified framework enables us to transfer results between models. For example, we generalize the binary model and the Hawkes process to the situation with synaptic conduction delays and simplify derivations for established results. Our approach is applicable to general network structures and suitable for the calculation of population averages. The derived averages are exact for fixed out-degree network architectures and approximate for fixed in-degree. We demonstrate how taking into account fluctuations in the linearization procedure increases the accuracy of the effective theory and we explain the class dependent differences between covariances in the time and the frequency domain. Finally we show that the oscillatory instability emerging in networks of LIF models with delayed inhibitory feedback is a model-invariant feature: the same structure of poles in the complex frequency plane determines the population power spectra.

## 1. Introduction

The meaning of correlated neural activity for the processing and representation of information in cortical networks is still not understood, but evidence for a pivotal role of correlations increases (recently reviewed in Cohen and Kohn, 2011). Different studies have shown that correlations can either decrease (Zohary et al., 1994) or increase (Sompolinsky et al., 2001) the signal to noise ratio of population signals, depending on the readout mechanism. The architecture of cortical networks is dominated by convergent and divergent connections among the neurons (Braitenberg and Schüz, 1991) causing correlated neuronal activity by common input from shared afferent neurons in addition to direct connections between pairs of neurons and common external signals. It has been shown that correlated activity can faithfully propagate through convergent-divergent feed forward structures, such as synfire chains (Abeles, 1991; Diesmann et al., 1999), a potential mechanism to convey signals in the brain. Correlated firing was also proposed as a key to the solution of the binding problem (von der Malsburg, 1981; Bienenstock, 1995; Singer, 1999), an idea that has been discussed controversially (Shadlen and Movshon, 1999). Independent of a direct functional role of correlations in cortical processing, the covariance function between the spiking activity of a pair of neurons contains the information about time intervals between spikes. Changes of synaptic coupling, mediated by spike-timing dependent synaptic plasticity (STDP, Markram et al., 1997; Bi and Poo, 1999), are hence sensitive to correlations. Understanding covariances in spiking networks is thus a prerequisite to investigate the evolution of synapses in plastic networks (Burkitt et al., 2007; Gilson et al., 2009, 2010).

On the other side, there is ubiquitous experimental evidence of correlated spike events in biological neural networks, going back to early reports on multi-unit recordings in cat auditory cortex (Perkel et al., 1967; Gerstein and Perkel, 1969), the observation of closely time-locked spikes appearing at behaviorally relevant points in time (Kilavik et al., 2009; Ito et al., 2011) and collective oscillations in cortex [recently reviewed in Buzsáki and Wang (2012)].

The existing theories explaining correlated activity use a multitude of different neuron models. Hawkes (1971) developed the theory of covariances for linear spiking Poisson neurons (Hawkes processes). Ginzburg and Sompolinsky (1994) presented the approach of linearization to treat fluctuations around the point of stationary activity and to obtain the covariances for networks of non-linear binary neurons. The formal concept of linearization allowed Brunel and Hakim (1999) and Brunel (2000) to explain fast collective gamma oscillations in networks of spiking leaky integrate-and-fire (LIF) neurons. Correlations in feed-forward networks of LIF models are studied in Moreno-Bote and Parga (2006), exact analytical solutions for such network architectures are given in Rosenbaum and Josic (2011) for the case of stochastic random walk models, and threshold crossing neuron models are considered in Tchumatchenko et al. (2010) and Burak et al. (2009). Covariances in structured networks are investigated for Hawkes processes (Pernice et al., 2011), and in linear approximation for LIF (Pernice et al., 2012) and exponential integrate-and-fire neurons (Trousdale et al., 2012). The latter three works employ an expansion of the propagator (time evolution operator) in terms of the order of interaction. Finally Buice et al. (2009) investigate higher order cumulants of the joint activity in networks of binary model neurons.

**Figure 1:**
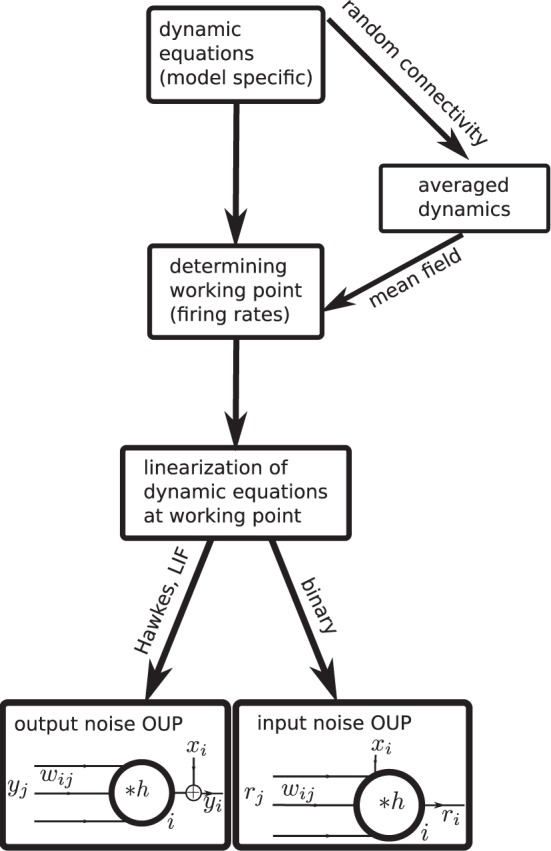
**Mapping different descriptions of neuronal dynamics to linear rate models (LRM)**. The arrows indicate analytical methods which enable a mapping from the original spiking (LIF model, Hawkes model) or binary neuron dynamics to the analytically more tractable linear rate models. Depending on the original dynamics (spiking or binary) the resulting LRM contains an additive noise component *x* either on the output side (left) or on the input side (right).

Analytical insight into a neuroscientific phenomenon based on correlated neuronal activity often requires a careful choice of the neuron model to arrive at a solvable problem. Hence a diversity of models has been proposed and is in use. This raises the question which features of covariances are generic properties of recurrent networks and which are specific to a certain model. Only if this question can be answered one can be sure that a particular result is not an artifact of oversimplified neuronal dynamics. Currently it is unclear how different neuron models relate to each other and whether and how results obtained with one model carry over to another. In this work we present a unified theoretical view on pairwise correlations in recurrent networks in the asynchronous and collective-oscillatory regime, approximating the response of different models to linear order. The joint treatment allows us to answer the question of genericness and moreover naturally leads to a classification of the considered models into only two categories, as illustrated in Figure 1. The classification in addition enables us to extend existing theoretical results to biologically relevant parameters, such as synaptic delays and the presence of inhibition, and to derive explicit expressions for the time-dependent covariance functions, in quantitative agreement with direct simulations, which can serve as a starting point for further work.

The remainder of this article is organized as follows. In the first part of our results in “Covariance structure of noisy rate models” we investigate the activity and the structure of covariance functions for two versions of linear rate models (LRM); one with input the other with output noise. If the activity relaxes exponentially after application of a short perturbation, both models coincide with the OUP. We mainly consider the latter case, although most results hold for arbitrary kernel functions. We extend the analytical solutions for the covariances in networks of OUP (Risken, 1996) to the neuroscientifically important case of synaptic conduction delays. Solutions are derived first for general forms of connectivity in “Solution of the convolution equation with input noise” for input noise and in “Solution of convolution equation with output noise” for output noise. After analyzing the spectral properties of the dynamics in the frequency domain in “Spectrum of the dynamics,” identifying poles of the propagators and their relation to collective oscillations in neuronal networks, we show in “Population-averaged covariances”' how to obtain pairwise averaged covariances in homogeneous Erdös-Rényi random networks. We explain in detail the use of the residue theorem to perform the Fourier back-transformation of covariance functions to the time domain in “Fourier back transformation” for general connectivity and in “Explicit expression for the population averaged cross covariance in the time domain” for averaged covariance functions in random networks, which allows us to obtain explicit results and to discuss class dependent features of covariance functions.

In the second part of our results in “Binary neurons,” “Hawkes processes,” and “Leaky integrate-and-fire neurons” we consider the mapping of different neuronal dynamics on either of the two flavors of the linear rate models discussed in the first part. The mapping procedure is qualitatively the same for all dynamics as illustrated in Figure 1: Starting from the dynamic equations of the respective model, we first determine the working point described in terms of the mean activity in the network. For unstructured homogeneous random networks this amounts to a mean-field description in terms of the population averaged activity (i.e., firing rate in spiking models). In the next step, a linearization of the dynamical equations is performed around this working point. We explain how fluctuations can be considered in the linearization procedure to improve its accuracy and we show how the effective linear dynamics maps to the LRM. We illustrate the results throughout by a quantitative comparison of the analytical results to direct numerical simulations of the original non-linear dynamics. The appendices “Implementation of noisy rate models,” “Implementation of binary neurons in a spiking simulator code,” and “Implementation of Hawkes neurons in a spiking simulator code.” describe the model implementations and are modules of our long-term collaborative project to provide the technology for neural systems simulations (Gewaltig and Diesmann, 2007).

## 2. Covariance structure of noisy rate models

### 2.1. Definition of models

Let us consider a network of linear model neurons, each characterized by a continuous fluctuating rate *r* and connections from neuron *j* to neuron *i* given by the element *w*_*ij*_ of the connectivity matrix **w**. We assume that the response of neuron *i* to input can be described by a linear kernel *h* so that the activity in the network fulfills

(1) r(t)=h(◦)∗[wr(◦−d)+bx(◦)](t),

where *f*(◦ − *d*) denotes the function *f* shifted by the delay *d*, **x** is an uncorrelated noise with

(2) $$\langle x_{i}(t)\rangle =0,\;\langle x_{i}(s)x_{j}(t)\rangle =\delta_{ij}\delta (s-t)\rho^{2}\;,$$

e.g., a Gaussian white noise and (*f* * *g*)(*t*) = ∫^*t*^_− ∞_*f*(*t* − *t*′) *g*(*t*′)*dt*′ is the convolution. With the particular choice ***b*** = **w**δ(◦ − *d*)* we obtain

(3) r(t)=[h(◦)∗w(r(◦−d)+x(◦−d))](t).

We call the dynamics (3) the linear noisy rate model (LRM) with noise applied to output, as the sum *r* + *x* appears on the right hand side. Alternatively, choosing ***b*** = **1** we define the model with input noise as

(4) r(t)=h(◦)∗[wr(◦−d)+x(◦)](t).

Hence, Equations (3) and (4) are special cases of (1). In the following we consider the particular case of an exponential kernel

(5) $$h(s)=\frac{1}{\tau }\theta (s)e^{-s/\tau },$$

where θ denotes the Heaviside function, θ(*t*) = 1 for *t* > 0, 0 else. Applying to (1) the operator $O=\tau \frac{d}{ds}+1$ which has *h* as a Green's function (i.e., *Oh* = δ) we get

(6) $$\tau \frac{d}{dt}r(t)+r(t)=wr(t-d)+bx(t),$$

which is the equation describing a set of delay coupled Ornstein-Uhlenbeck-processes (OUP) with input or output noise for ***b*** = **1** or ***b*** = **w**δ(◦ − *d*)*, respectively. We use this representation in “Binary neurons” to show the correspondence to networks of binary neurons.

### 2.2. Solution of the convolution equation with input noise

The solution for the system with input noise obtained from the definition (4) after Fourier transformation is

(7) $$R=H_{d}wR+HX,$$

where the delay is consumed by the kernel function $h_{d}(s)=\frac{1}{\tau }\theta (s-d)e^{-(s-d)/\tau }$. We use capital letters throughout the text to denote objects in the Fourier domain and lower case letters for objects in the time domain. Solved for **R** = (1 − *H*_*d*_**w**)^−1^*H***X** the covariance function of **r** in the Fourier domain is found with the Wiener–Khinchin theorem (Gardiner, 2004) as 〈**R**(ω)**R**^*T*^(−ω)〉, also called the cross spectrum

(8) $$\begin{array}{c} \begin{array}{l} C(\omega )=\langle R(\omega )R^{T}(-\omega )\rangle \\ \;={\left(1-H_{d}\left(\omega \right)w\right)}^{-1}H(\omega )\langle X(\omega )X^{T}(-\omega )\rangle \\ \;H(-\omega ){\left(1-H_{d}\left(-\omega \right)w^{T}\right)}^{-1}\\ \;={\left(H_{d}{\left(\omega \right)}^{-1}-w\right)}^{-1}D{\left(H_{d}{\left(-\omega \right)}^{-1}-w^{T}\right)}^{-1}, \end{array} \end{array}$$

where we introduced the matrix **D** = 〈**X**(ω)**X**^*T*^(−ω)〉. From the second to the third line we used the fact that the non-delayed kernels *H*(ω) can be replaced by delayed kernels *H*_*d*_(ω) and that the corresponding phase factors *e*^*i*ω*d*^ and *e*^− *i*ω*d*^ cancel each other. If **x** is a vector of pairwise uncorrelated noise, **D** is a diagonal matrix and needs to be chosen accordingly in order for the cross spectrum (8) to coincide (neglecting non-linear effects) with the cross spectrum of a network of binary neurons, as described in “Equivalence of binary neurons and Ornstein–Uhlenbeck processes”.

### 2.3. Solution of convolution equation with output noise

For the system with output noise we consider the quantity *y*_*i*_ = *r*_*i*_ + *x*_*i*_ as the dynamic variable representing the activity of neuron *i* and aim to determine pairwise correlations. It is easy to get from (3) after Fourier transformation

(9) $$R=H_{d}w(R+X),$$

which can be solved for **R** = (1 − *H*_*d*_**w**)^−1^*H*_*d*_**wX** in order to determine the Fourier transform of **Y** as

(10) $$Y=R+X={\left(1-H_{d}w\right)}^{-1}X.$$

The cross spectrum hence follows as

(11) $$\begin{array}{c} \begin{array}{l} C(\omega )=\langle Y(\omega )Y^{T}(-\omega )\rangle \\ \;={\left(1-H_{d}\left(\omega \right)w\right)}^{-1}\langle X(\omega )X^{T}(-\omega )\rangle {\left(1-H_{d}\left(-\omega \right)w^{T}\right)}^{-1}\\ \;={\left(1-H_{d}\left(\omega \right)w\right)}^{-1}D{\left(1-H_{d}\left(-\omega \right)w^{T}\right)}^{-1}, \end{array} \end{array}$$

with **D** = 〈**X**(ω)**X**^*T*^(−ω)〉. **D** is a diagonal matrix with the *i*-th diagonal entry ρ^2^_*i*_. For the correspondence to spiking models **D** must be chosen appropriately, as discussed in “Hawkes processes” and “Leaky integrate-and-fire neurons” for Hawkes processes and LIF neurons, respectively.

### 2.4. Spectrum of the dynamics

For both linear rate dynamics, with output and with input noise, the cross spectrum **C**(ω) has poles at certain frequencies ω in the complex plane. These poles are defined by the zeros of det(*H*_*d*_(ω)^−1^ − **w**) and the corresponding term with the opposite sign of ω. The zeros of det(*H*_*d*_(ω)^− 1^ − **w**) are solutions of the equation

$$H_{d}{\left(\omega \right)}^{-1}=(1+i\omega \tau )e^{i\omega d}=L_{j}$$

where *L*_*j*_ is the *j*-th eigenvalue of **w**. The same set of poles arises from (1) when solving for **R**. For *d* > 0 and the exponential kernel (5), the poles can be expressed as

(12) $$z_{k}(L_{j})=\frac{i}{\tau }-\frac{i}{d}W_{k}\left(L_{j}\frac{d}{\tau }e^{\frac{d}{\tau }}\right),$$

where *W*_*k*_ is the *k*-th of the infinitely many branches of the Lambert-W function (Corless et al., 1996). For vanishing synaptic delay *d* = 0 there is obviously only one solution for every *L*_*j*_ given by $z=\frac{-i}{\tau }(L_{j}-1)$.

**Figure 2:**
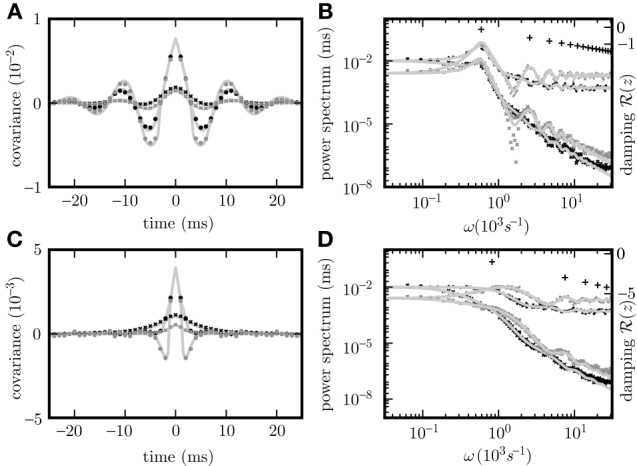
**Pole structure determines dynamics**. Autocovariance of the population activity **(A,C)** measured in ρ^2^/τ and its Fourier transform called power spectrum **(B,D)** of the rate models with output noise (dots) (3) and input noise (diagonal crosses) (4) for delays *d* = 3 ms **(A,B)**, and *d* = 1 ms **(C,D)**. Black symbols show averages over the excitatory population activity and gray symbols over the inhibitory activity obtained by direct simulation. Light gray curves show theoretical predictions for the spectrum (20) and the covariance (21) for output noise and the spectrum (17) and the covariance (18) for input noise. Black crosses (12) in **(B,D)** denote the locations of the poles of the cross spectra - with the real parts corresponding to the damping (vertical axis), and the imaginary parts to oscillation frequencies (horizontal axis). The detailed parameters for this and following figures are given in “Parameters of simulations”.

Given the same parameters *d*, **w**, τ, the pole structures of the cross spectra of both systems (8) and (11) are identical, since the former can be obtained from the latter by multiplication with (*H*_*d*_(ω)*H*_*d*_(−ω))^−1^ = (*H*(ω)*H*(−ω))^−1^, which has no poles. The only exception causing a different pole structure for the two models is the existence of an eigenvalue *L*_*j*_ = 0 of the connectivity matrix **w**, corresponding to a pole $z(0)=\frac{i}{\tau }$. However, this pole corresponds to an exponential decay of the covariance for input noise in the time domain and hence does not contribute to oscillations. For output noise, the multiplication with the term (*H*(ω)*H*(−ω))^−1^, vanishing at $\omega =\frac{i}{\tau }$, cancels this pole in the covariance. Consequently both dynamics exhibit similar oscillations. A typical spectrum of poles for a negative eigenvalue *L*_*j*_ < 0 is shown in Figures 2B,D.

### 2.5. Population-averaged covariances

Often it is desirable to consider not the whole covariance matrix but averages over subpopulations of pairs of neurons. For instance the average over the whole network would result in a single scalar value. Separately averaging pairs, distinguishing excitatory and inhibitory neuron populations, yields a 2 by 2 matrix of covariances. For these simpler objects closed form solutions can be obtained, which already preserve some useful information and show important features of the network. Averaged covariances are also useful for comparison with simulations and experimental results.

In the following we consider a recurrent random network of *N*_*e*_ = *N* excitatory and *N*_*i*_ = γ*N* inhibitory neurons with synaptic weight *w* for excitatory and −*gw* for inhibitory synapses. The probability *p* determines the existence of a connection between two randomly chosen neurons. We study the dynamics averaged over the two subpopulations by introducing the quantities $r_{a}=\frac{1}{N_{a}}\sum_{j\;\in \;a}r_{j}$ and noise terms $x_{a}=\frac{1}{N_{a}}\sum_{j\;\in \;a}x_{j}$ for *a* ∈ {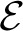, 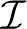}; indices 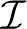 and 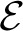 stand for inhibitory and excitatory neurons and corresponding quantities. Calculating the average local input *N*^−1^_*a*_∑_*j* ∈ *a*_*w*_*jk*_*r*_*k*_ to a neuron of type *a*, we obtain 
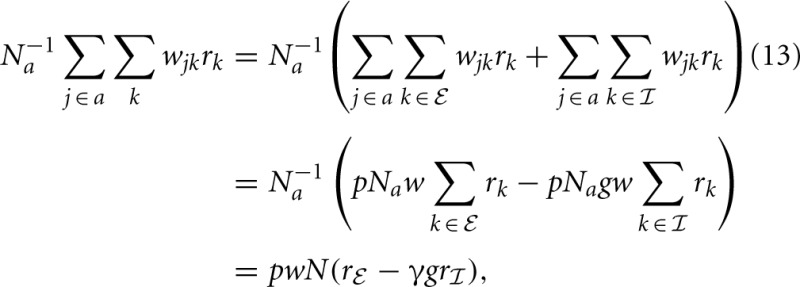
 where, from the second to the third line we used the fact that in expectation a given neuron *k* has *pN*_*a*_ targets in the population *a*. The reduction to the averaged system in (13) is exact if in every column *k* in **w**_*jk*_ there are exactly *K* non-zero elements for *j* ∈ 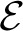 and γ*K* for *j* ∈ 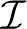, which is the case for networks with fixed out-degree (number of outgoing connections of a neuron to the neurons of a particular type is kept constant), as noted earlier (Tetzlaff et al., 2012). For fixed in-degree (number of connections to a neuron coming in from the neurons of a particular type is kept constant) the substitution of *r*_*j* ∈ *a*_ by *r*_*a*_ is an additional approximation, which could be considered as an average over possible realizations of the random connectivity. In both cases the effective population-averaged connectivity matrix **M** turns out to be

(14) $$M=Kw\left(\begin{array}{c} \begin{array}{l} 1-\gamma g\\ 1-\gamma g \end{array} \end{array}\right)\text{​},$$

with *K* = *pN*. So the averaged activities fulfill the same Equations (3) and (4) with the non-averaged quantities **r**, **x**, and **w** replaced by their averaged counterparts **r** = (*r*_
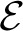
_, *r*_
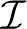
_)^*T*^, **x** = (*x*_
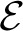
_, *x*_
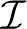
_)^*T*^, and **M**. The population averaged activities *r*_*a*_ are directly related to the block-wise averaged covariance matrix 
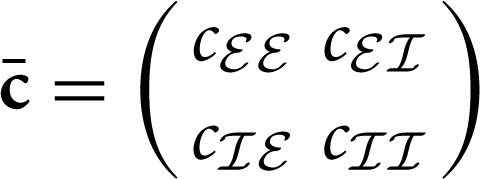
, with *c*_*ab*_ = *N*^−1^_*a*_*N*^−1^_*b*_∑_*i* ∈ *a*_∑_*j* ∈ *b*_ *c*_*ij*_. With

(15) $$\begin{array}{l} {\bar{D}}_{ab}=N_{a}^{-1}N_{b}^{-1}\langle \sum_{i\in a}x_{i}\sum_{j\in b}x_{j}\rangle \\ \;=N_{a}^{-1}N_{b}^{-1}\sum_{i\in a}\sum_{j\in b}D_{ij}\\ \;=\delta_{ab}N_{a}/N_{a}^{2}\rho^{2}=\delta_{ab}N_{a}^{-1}\rho^{2} \end{array}$$

we replace **D** by $\bar{D}=\rho^{2}\left(\begin{array}{cc} N^{-1} & 0\\ 0 & {\left(\gamma N\right)}^{-1} \end{array}\right)$ and **c** by **c** so that the same Equations (11) and (8) and their general solutions also hold for the block-wise averaged covariance matrices.

The covariance matrices separately averaged over pairs of excitatory, inhibitory or mixed pairs are shown in Figure 2 for both linear rate dynamics (3) and (4). (Parameters for all simulations presented in this article are collected in “Parameters of simulations,” the implementation of LRM is described in “Implementation of noisy rate models”). The poles of both models shown in Figure 2B are given by (12) and coincide with the peaks in the cross spectra (8) and (11) for output and input noise, respectively. The results of direct simulation and the theoretical prediction are shown for two different delays, with the longer delay leading to stronger oscillations.

**Figure 3:**
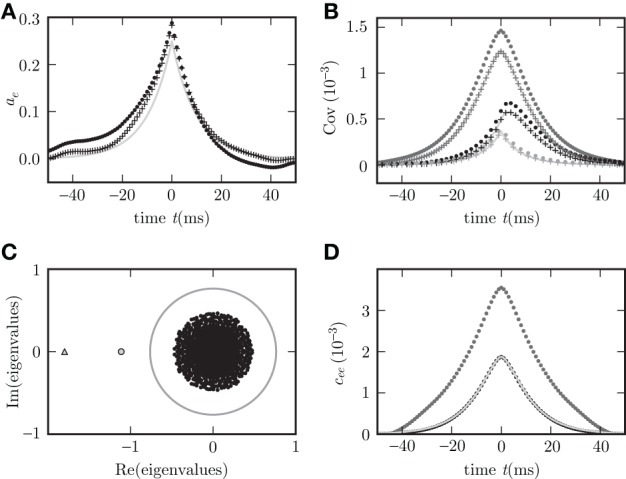
**Limits of the theory for fixed in-degree and fixed out-degree**. Autocovariance **(A)** and covariance **(B)** in random networks with fixed in-degree (dots) and fixed out-degree (crosses). Simulation results for *c*_
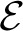

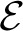
_, *c*_
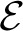

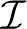
_, and *c*_
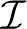

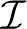
_ are shown in dark gray, black and light gray, respectively for synaptic weight *w* = 0.011 far from bifurcation. For larger synaptic weight *w* = 0.018 close to bifurcation (see text at the end of “Population-averaged covariances”), *c*_
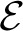

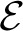
_ is also shown in **(D)** for fixed in-degree (dark gray dots) and for fixed out-degree (black dots). Corresponding theoretical predictions for the autocovariance (34) **(A)** and the covariance (18) **(B,D)** are plotted as light gray curves throughout. The set of eigenvalues is shown as black dots in panel **(C)** for the smaller weight. The gray circle denotes the spectral radius $w\sqrt{Np(1-p)(1+\gamma g^{2})}$ (Rajan and Abbott, 2006; Kriener et al., 2008) confining the set of eigenvalues for the larger weight. The small filled gray circle and the triangle show the effective eigenvalues *L* of the averaged systems for small and large weight, respectively.

Figure 3C shows the distribution of eigenvalues in the complex plane for two random connectivity matrices with different synaptic amplitudes *w*. The model exhibits a bifurcation, if at least one eigenvalue assumes a zero real part. For fixed out-degree the averaging procedure (13) is exact, reflected by the precise agreement of theory and simulation in Figure 3D. For fixed in-degree, the averaging procedure (13) is an approximation, which is good only for parameters far from the bifurcation. Even in this regime still small deviations of the theory from the simulation results are visible in Figure 3B. On the stable side close to a bifurcation, the appearance of long living modes causes large fluctuations. These weakly damped modes appearing in one particular realization of the connectivity matrix are not represented after the replacement of the full matrix **w** by the average **M** over matrix realizations. The eigenvalue spectrum of the connectivity matrix provides an alternative way to understand the deviations. By the averaging the set of *N* eigenvalues of the connectivity matrix is replaced withby the two eigenvalues of the reduced matrix **M**, one of which is zero due to identical rows of **M**. The eigenvalue spectrum of the full matrix is illustrated in Figure 3C. Even if the eigenvalue(s) *L*^**M**^ of **M** are far in the stable region (corresponding to 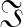
(*z*(*L*^**M**^)) > 0) some eigenvalues *L*^**w**^ of the full connectivity matrix in the vicinity of the bifurcation region may still have an imaginary part becoming negative and the system can feel their influence, shown in Figure 3D.

### 2.6. Fourier back transformation

Although the cross spectral matrices (8) and (11) for both dynamics look similar in the Fourier domain, the procedures for back transformation differ in detail. In both cases, the Fourier integral along the real ω-axis can be extended to a closed integration contour by a semi-circle with infinite radius centered at 0 in the appropriately chosen half-plane. The half-plane needs to be selected such that the contribution of the integration along the semi-circle vanishes. By employing the residue theorem (Bronstein et al., 1999) the integral can be replaced by a sum over residua of the poles encircled by the contour. For a general covariance matrix we only need to calculate **c**(*t*) for *t* ≥ 0, as for *t* < 0 the solution can be found by symmetry **c**(*t*) = **c**^*T*^(−*t*).

For input noise it is possible to close the contour in the upper half-plane where the integrand **C**(ω)*e*^*i*ω*t*^ vanishes for |ω| → ∞ for all *t* > 0, as |*C*_*ij*_(ω)| decays as |ω|^−2^. This can be seen from (8), because the highest order of *H*^−1^_*d*_ ∝ ω appearing in det(*H*^−1^_*d*_ − **w**) is equal to the dimensionality *N* of **w** (*N* = 2 for **M**), and in det(adjugate matrix *ij* of *H*^−1^_*d*_ − **w**) it is *N* − 1 (*i* = *j*) or *N* − 2 (*i* ≠ *j*). So |(*H*^−1^_*d*_ − **w**)^−1^| is proportional to |ω|^−1^|*e*^− *i*ω*d*^| and |**C**(ω)|∝|ω|^−2^ for large |ω|.

For the case of output noise (11) **C**(ω) can be obtained from the **C**(ω) for input noise (8) multiplied with (*H*_*d*_(ω)*H*_*d*_(−ω))^−1^ ~ |ω|^2^ for large |ω|. The multiplication with this factor changes the asymptotic behavior of the integrand, which therefore contains terms converging to a constant value and terms decaying like |ω|^−1^ for |ω| → ∞. These terms result in non-vanishing integrals over the semicircle in the upper half-plane and have to be considered separately. To this end we rewrite (11) as

(16) $$\begin{array}{cc} \begin{array}{l} C\left(\omega \right)=\left({\left(1-H_{d}\left(\omega \right)w\right)}^{-1}H_{d}\left(\omega \right)w+1\right)\\ \;D\left(w^{T}H_{d}\left(-\omega \right){\left(1-H_{d}\left(-\omega \right)w^{T}\right)}^{-1}+1\right)\\ \;={\left(1-H_{d}\left(\omega \right)w\right)}^{-1}H_{d}\left(\omega \right)wDw^{T}H_{d}\left(-\omega \right){\left(1-H_{d}\left(-\omega \right)w^{T}\right)}^{-1}\\ \;+{\left(1-H_{d}\left(\omega \right)w\right)}^{-1}H_{d}\left(\omega \right)wD\\ \;+Dw^{T}H_{d}\left(-\omega \right){\left(1-H_{d}\left(-\omega \right)w^{T}\right)}^{-1}\\ \;+D, \end{array} & \left(16\right) \end{array}$$

and find the constant term **D** which turns into a δ-function in the time domain. The first term in the second line of (16) decays like |ω|^−2^ and can be transformed just as **C**(ω) for input noise closing the contour in the upper half-plane. The second and third term are the transposed complex conjugates of each other, because of the dependence of *H* on −ω instead of ω, and require a special consideration. Multiplied by *e*^*i*ω*t*^ under the Fourier integral, the first term is proportional to *H*_*d*_*e*^*i*ω*t*^ ~ ω^−1^*e*^*i*ω(*t* − *d*)^ and vanishes faster than |ω|^−1^ for large |ω| in the upper half-plane for *t* > *d* and in the lower half plane for *t* < *d*. For the second term the half planes are interchanged. The application of the residue theorem requires closing the integration contour in the half-plane where the integral over the semi-circle vanishes faster than |ω|^−1^. For **w** = **M** and in the general case of a stable dynamics all poles of the first term are in the upper half-plane 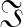
(*z*_*k*_(*L*_*j*_)) > 0, and have no contribution to **c**(*t*) for *t* < *d*. For the second term the same is true for *t* > −*d*; these terms correspond to the jumps of **c**(*t*) after one delay, caused by the effect of the sending neuron arriving at the other neurons in the system after one synaptic delay. These terms correspond to the response of the system to the impulse of the sending neuron – hence we call them “echo terms” in the following (Helias et al., 2013). The presence of such discontinuous jumps at time points *d* and −*d* in the case of output noise is reflected in the convolution of *h***w** with **D** in the time domain in (37). For input noise the absence of discontinuities can be inferred from the absence of such terms in (33), where the derivative of the correlation function is equal to the sum of finite terms. The first summand in (16) corresponds to the covariance evoked by fluctuations propagating through the system originating from the same neuron and we call it “correlated input term”. In the system with input noise a similar separation into effective echo and correlated input terms can be performed. We obtain the correlated input term as the covariance in an auxiliary population without outgoing connections and echo terms as the difference between the full covariance between neurons within the network and the correlated input term.

### 2.7. Explicit expression for the population averaged cross covariance in the time domain

We obtain the population averaged cross spectrum in a recurrent random network of Ornstein–Uhlenbeck processes (OUP) with input noise by inserting the averaged connectivity matrix **w** = **M** (14) into (8). The explicit expression for the covariance function follows by taking into account all (both) eigenvalues of **M** with values 0 and *L* = *Kw*(1 − γ*g*). The detailed derivation of the results presented in this section are documented in “Calculation of the Population Averaged Cross Covariance in Time Domain”. The expression for the cross spectrum (8) takes the form

(17) $$\begin{array}{l} C(\omega )=f(\omega )f(-\omega )\left(1+Kw\left(\begin{array}{c} \begin{array}{cc} \gamma g & -\gamma g\\ 1 & -1 \end{array} \end{array}\right)H_{d}(\omega )\right)\\ \;D\left(1+Kw\left(\begin{array}{cc} \gamma g & 1\\ -\gamma g & -1 \end{array}\right)H_{d}(-\omega )\right), \end{array}$$

where we introduced *f*(ω) = (*H*_*d*_(ω)^−1^ − *L*)^−1^ as a short hand. Sorting the terms by their dependence on ω, introducing the functions Φ_1_(ω),…,Φ_4_(ω) for this dependence, and φ_1_(*t*),…,φ_4_(*t*) for the corresponding functions in the time domain, the covariance in the time domain $c(t)=\frac{1}{2\pi }\int_{-\infty }^{+\infty }C(\omega )e^{i\omega t}d\omega$ takes the form

$$\begin{array}{l} c(t)=D\varphi_{1}(t)\\ \;+Kw\left(\left(\begin{array}{cc} \gamma g & -\gamma g\\ 1 & -1 \end{array}\right)D\varphi_{2}(t)+D\left(\begin{array}{cc} \gamma g & 1\\ -\gamma g & -1 \end{array}\right)\varphi_{3}(t)\right)\\ \;+K^{2}w^{2}\left(\begin{array}{c} \begin{array}{cc} \gamma g & -\gamma g\\ 1 & -1 \end{array} \end{array}\right)D\left(\begin{array}{cc} \gamma g & 1\\ -\gamma g & -1 \end{array}\right)\varphi_{4}(t). \end{array}$$

The previous expression is valid for arbitrary **D**. In simulations presented in this article we consider identical marginal input statistics for all neurons. In this case the averaged activities for excitatory and inhibitory neurons are the same, so we can insert the special form of **D** given in (15), which results in

(18) $$\begin{array}{l} c(t)=\frac{\rho^{2}}{N}\left(\begin{array}{cc} 1 & 0\\ 0 & \gamma^{-1} \end{array}\right)\varphi_{1}(t)\\ \;+\frac{\rho^{2}}{N}Kw\left(\begin{array}{cc} \gamma g & -g\\ 1 & -\gamma^{-1} \end{array}\right)\varphi_{2}(t)+\frac{\rho^{2}}{N}Kw\left(\begin{array}{cc} \gamma g & 1\\ -g & -\gamma^{-1} \end{array}\right)\varphi_{3}(t)\\ \;+\frac{\rho^{2}}{N}(\gamma +1)K^{2}w^{2}\left(\begin{array}{cc} \begin{array}{c} \gamma g^{2} \end{array} & g\\ g & -\gamma^{-1} \end{array}\right)\varphi_{4}(t). \end{array}$$

The time-dependent functions φ_1_,…,φ_4_ are the same in both cases. Using the residue theorem $\varphi_{i}(t)=\frac{1}{2\pi }\int_{-\infty }^{+\infty }\Phi_{i}(\omega )e^{i\omega t}d\omega =i\sum_{z\in \text{poles of }\Phi_{i}}\text{Res}(\Phi_{i},z)e^{izt}$ for *t* ≥ 0 they can be expressed as a sum over the poles *z*_*k*_(*L*) given by (12) and the pole $z=\frac{i}{\tau }$ of *H*_*d*_(ω). At ω = *z*_*k*_(*L*) the residue of *f*(ω) is Res(*f*, ω = *z*_*k*_(*L*)) = (*idL* + *i*τ*e*^*i*ω*d*^)^−1^, the residue of *H*_*d*_(ω) at $z=\frac{i}{\tau }$ is $-\frac{i}{\tau }e^{d/\tau }$, so that the explicit forms of φ_1_,…,φ_4_ follow as

(19) $$\begin{array}{l} \varphi_{1}(t)=\sum_{\omega =z_{k}(L)}i\text{Res}(f,\omega )f(-\omega )e^{i\omega t}\\ \varphi_{2}(t)=\sum_{\omega =z_{k}(L)}i\text{Res}(f,\omega )f(-\omega )H_{d}(\omega )e^{i\omega t}\\ \;+\frac{e^{(d-t)/\tau }}{\tau }f\left(\frac{i}{\tau }\right)f\left(-\frac{i}{\tau }\right)\\ \varphi_{3}(t)=\sum_{\omega =z_{k}(L)}i\text{Res}(f,\omega )f(-\omega )H_{d}(-\omega )e^{i\omega t}\\ \varphi_{4}(t)=\sum_{\omega =z_{k}(L)}i\text{Res}(f,\omega )f(-\omega )H_{d}(\omega )H_{d}(-\omega )e^{i\omega t}\\ \;+\frac{e^{-t/\tau }}{2\tau }f\left(\frac{i}{\tau }\right)f\left(-\frac{i}{\tau }\right). \end{array}$$

The corresponding expression for **C**(ω) for output noise is obtained by multiplying (17) with *H*^−1^_*d*_(ω)*H*^−1^_*d*_(−ω) = (1 + ω^2^τ^2^)

(20) $$\begin{array}{l} \text{​​}C(\omega )=H_{d}^{-1}(\omega )H_{d}^{-1}(-\omega )f(\omega )f(-\omega )\\ \;\times (1+Kw\left(\begin{array}{cc} \gamma g & -\gamma g\\ 1 & -1 \end{array}\right)H_{d}(\omega ))D(1+Kw\left(\begin{array}{cc} \gamma g & 1\\ -\gamma g & -1 \end{array}\right)H_{d}(-\omega )), \end{array}$$

which, after Fourier transform, provides the expression for **c**(*t*) in the time domain for *t* ≥ 0

(21) $$\begin{array}{l} c(t)=MDM^{T}\varphi_{1}(t)+MD\varphi_{0}(t)+D\delta (t)\\ \;=K^{2}w^{2}\frac{\rho^{2}}{N}(1+\gamma g^{2})\left(\begin{array}{cc} 1 & 1\\ 1 & 1 \end{array}\right)\varphi_{1}(t)+Kw\frac{\rho^{2}}{N}\left(\begin{array}{cc} 1 & -g\\ 1 & -g \end{array}\right)\varphi_{0}(t)+\frac{\rho^{2}}{N}\left(\begin{array}{cc} 1 & 0\\ 0 & \gamma^{-1} \end{array}\right)\delta (t). \end{array}$$

As in (18), the first line holds for arbitrary **D**, and the second for **D** given by (15), valid if the firing rates are homogeneous. φ_1_ is defined as before, and

(22) $$\varphi_{0}(t)=\theta (t-d)\sum_{\omega =z_{k}(L)}{\left(dL+\tau e^{i\omega d}\right)}^{-1}e^{i\omega t}$$

vanishes for *t* < *d*. All matrix elements of the first term in (21) are identical. Therefore all elements of **c**(*t*) are equal for 0 < |*t*| < *d*. Both rows of the matrix in front of φ_0_ are identical, so for *t* > 0 the off diagonal term *c*_
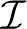

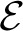
_ coincides with *c*_
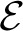

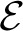
_ and *c*_
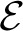

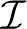
_ with *c*_
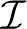

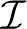
_ and vice versa for *t* < 0.

**Figure 4:**
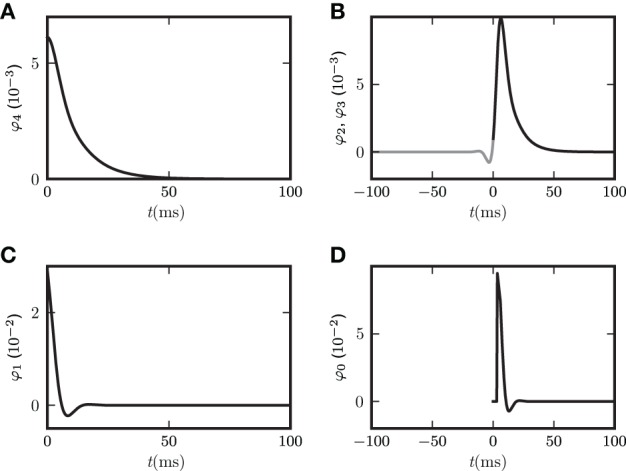
**Functions φ_0_ (D), φ_1_ (C), φ_2_, φ_3_ (B), φ_4_ (A) introduced in (19) and (22) for decomposition of covariance **c**(*t*)**. In **(B)** φ_3_(−*t*) is shown in gray and φ_2_(*t*) in black. The two functions are continuations of each other, joint at *t* = 0. Both functions appear in the echo term for input noise. The function φ_0_ in **(D)** describing the corresponding echo term in the case of output noise is shifted to be aligned with the function in **(B)** to facilitate the comparison of **(B,D)**. Parameters in all panels are *d* = 3 ms, τ = 10 ms, *L* = −1.72.

As an illustration we show the functions φ_0_,…,φ_4_ for one set of parameters in Figure 4. The left panels **(A,C)** correspond to contributions to the covariance caused by common input to a pair of neurons, the right panels **(B,D)** to terms due to the effect of one of the neurons' activities on the remaining network (echo terms). The upper panels **(A,B)** belong to the model with input noise, the lower panel **(C,D)** to the one with output noise.

For the rate dynamics with output noise, the term with φ_1_ in (21) (shown in Figure 4C) is symmetric and describes the common input covariance and the term with φ_0_ (shown in Figure 4D) is the echo part of the covariance. For the rate dynamics with input noise (18) the term containing φ_4_ (shown in Figure 4A) is caused by common input and is hence also symmetric, the terms with φ_2_ and φ_3_ (shown in Figure 4B) correspond to the echo part and have hence their peak outside the origin. The second echo term in (18) is equal to the first one transposed and with opposite sign of the time argument, so we show φ_2_(*t*) and φ_3_(−*t*) together in one panel in Figure 4B. Note that for input noise, the term with φ_1_ describes the autocovariance, which corresponds to the term with the δ-function in case of output noise.

The solution (18) is visualized in Figure 6, the solution (21) in Figure 7, and the decomposition into common input and echo parts is also shown and compared to direct simulations in Figure 8.

## 3. Binary neurons

In the following sections we study, in turn, the binary neuron model, the Hawkes model and the LIF model and show how they can be mapped to one of the two OUPs; either the one with input or the one with output noise, so that the explicit solutions (18) and (21) for the covariances derived in the previous section can be applied. In the present section, we start with the binary neuron model (Ginzburg and Sompolinsky, 1994; Buice et al., 2009).

Following Ginzburg and Sompolinsky (1994) the state of the network of *N* binary model neurons is described by a binary vector **n** ∈ {0, 1}^*N*^ and each neuron is updated at independently drawn time points with exponentially distributed intervals of mean duration τ. This stochastic update constitutes a source of noise in the system. Given the *i*-th neuron is updated, the probability to end in the up-state (*n*_*i*_ = 1) is determined by the gain function *F*_*i*_(**n**) which depends on the activity **n** of all other neurons. The probability to end in the down state (*n*_*i*_ = 0) is 1 − *F*_*i*_(**n**). Here we implemented the binary model in the NEST simulator (Gewaltig and Diesmann, 2007) as described in “Implementation of Binary Neurons in a Spiking Simulator~Code”. Such systems have been considered earlier (Ginzburg and Sompolinsky, 1994; Buice et al., 2009), and here we follow the notation employed in the latter work. In the following we collect results that have been derived in these works and refer the reader to these publications for the details of the derivations. The zero-time lag covariance function is defined as *c*_*ij*_(*t*) = 〈*n*_*i*_(*t*)*n*_*j*_(*t*)〉 − *a*_*i*_(*t*)*a*_*j*_(*t*), with the expectation value 〈〉 taken over different realizations of the stochastic dynamics. Here **a**(*t*) = (*a*_1_(*t*),…,*a*_*N*_(*t*))^*T*^ is the vector of mean activities *a*_*i*_(*t*) = 〈*n*_*i*_(*t*)〉. *c*_*ij*_(*t*) fulfills the differential equation

$$\begin{array}{l} \tau \frac{d}{dt}c_{ij}(t)=-2c_{ij}(t)+\langle (n_{j}(t)-a_{j}(t))F_{i}(n)\rangle \\ \;+\langle (n_{i}(t)-a_{i}(t))F_{j}(n)\rangle . \end{array}$$

In the stationary state, the covariance therefore fulfills

(23) $$c_{ij}=\frac{1}{2}\langle (n_{j}-a_{j})F_{i}(n)\rangle +\frac{1}{2}\langle (n_{i}-a_{i})F_{j}(n)\rangle .$$

The time lagged covariance *c*_*ij*_(*t, s*) = 〈*n*_*i*_(*t*)*n*_*j*_(*s*)〉 − *a*_*i*_(*t*)*a*_*j*_(*s*) fulfills for *t* > *s* the differential equation

(24) $$\tau \frac{d}{dt}c_{ij}(t,s)=-c_{ij}(t,s)+\langle F_{i}(n,t)(n_{j}(s)-a_{j}(s))\rangle .$$

This equation is also true for *i* = *j*, the autocovariance. The term 〈*F*_*i*_(**n**, *t*)(*n*_*j*_(*s*) − *a*_*j*_(*s*))〉 has a simple interpretation: it measures the influence of a fluctuation of neuron *j* at time *s* around its mean value on the gain of neuron *i* at time *t* (Ginzburg and Sompolinsky, 1994). We now assume a particular form for the coupling between neurons

(25) $$F_{i}(n,t)=\phi (J_{i}n(t-d))=\phi \left(\sum_{k=1}^{N}J_{ik}n_{k}(t-d)\right),$$

where **J**_*i*_ is the vector of incoming synaptic weights into neuron *i* and ϕ is a non-linear gain function. Assuming that the fluctuations of the total input **J**_*i*_**n** into the *i*-th neuron are sufficiently small to allow a linearization of the gain function ϕ, we obtain the Taylor expansion

$$F_{i}(n,t)=F_{i}(a)+\phi^{\prime }(J_{i}a)J_{i}(n(t-d)-a(t-d)),$$

where

(26) $$\phi^{\prime }(J_{i}a)$$

is the slope of the gain function at the point of mean input.

Up to this point the treatment of the system is identical to the work of Ginzburg and Sompolinsky (1994). Now we present an alternative approach for the linearization which takes into account the effect of fluctuations in the input. For sufficiently asynchronous network states, the fluctuations in the input **J**_*i*_**n**(*t* − *d*) to neuron *i* can be approximated by a Gaussian distribution 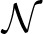
(μ, σ). In the following we consider a homogeneous random network with fixed in-degree as described in “Population-averaged covariances”. As each neuron receives the same number *K* of excitatory and γ*K* inhibitory synapses, the marginal statistics of the summed input to each neuron is identical. The mean input to a neuron then is μ = *KJ*(1 − γ*g*)*a*, where *a* is the mean activity of a neuron in the network. If correlations are small, the variance of this input signal distribution can be approximated as the sum of the variances of the individual contributions from the incoming signals, resulting in σ^2^ = *KJ*^2^(1 + γ*g*^2^)*a*(1 − *a*), where we used the fact that the variance of a binary variable with mean *a* is *a*(1 − *a*). This results from a direct calculation: since *n* ∈ {0, 1}, *n*^2^ = *n*, so that the variance is 〈*n*^2^〉 − 〈*n*〉^2^ = 〈*n*〉 − 〈*n*〉^2^ = *a*(1 − *a*). Averaging the slope ϕ′ of the gain function over the distribution of the input variable results in the averaged slope 
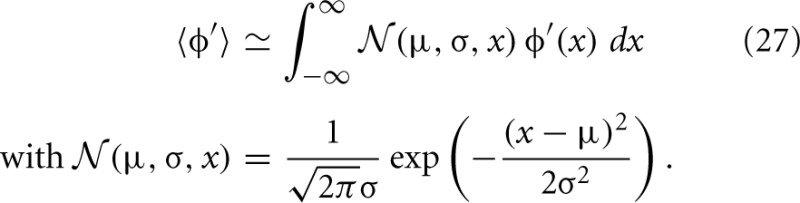


**Figure 5:**
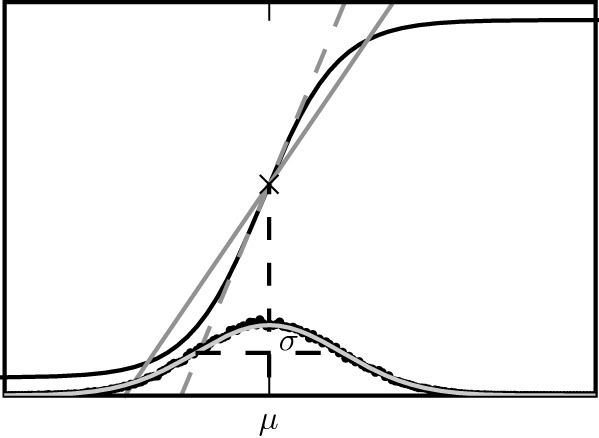
**Alternative linearizations of the binary neuron model**. The black curve represents the non-linear gain function $\phi (x)=\frac{1}{2}+\frac{1}{2}$ tanh(β*x*). The dashed gray line is its tangent at the mean input value (denoted by the diagonal cross). The solid curve is the slope 〈ϕ′〉 averaged over the distribution of the fluctuating input (27). This distribution estimated from direct simulation is presented by black dots, the corresponding theoretical prediction of a normal distribution 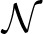
(μ,σ) (27) is shown as the light gray curve.

**Figure 6:**
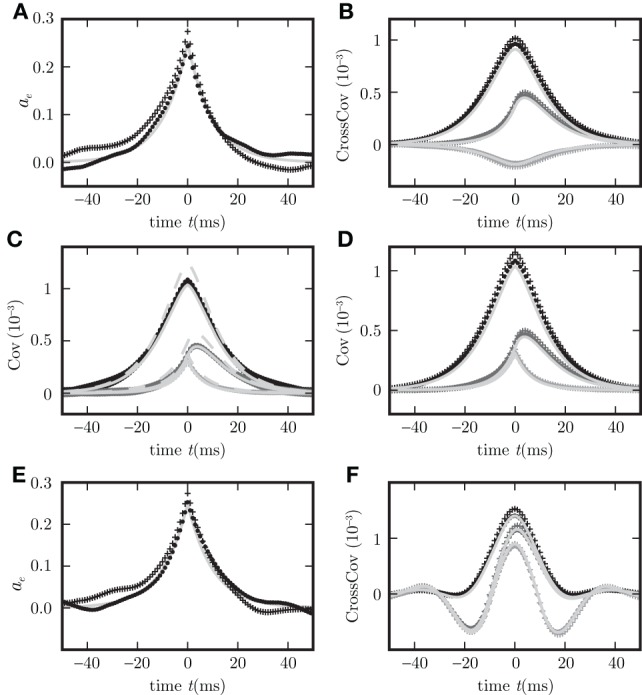
**Binary model neuron corresponds to OUP model with input noise**. Autocovariance **(A)**, crosscovarince **(B)**, and autocovariance of population averaged activity **(C,D)**, for binary neurons (dots) and rate model with input noise (crosses). *c*_
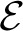

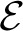
_, *c*_
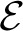

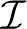
_ and *c*_
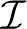

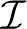
_ are shown in black, gray, and light gray. Corresponding theoretical predictions (18) in **(C,D)**, (34) in **(A)**, their difference in **(C)** are plotted as light gray curves throughout. Dashed curve in **(C)** represents the theoretical prediction using the linearization with the slope at the mean activity (26), the solid curve shows the results for the slope averaged over Gaussian distributed input fluctuations (27). The spread of the simulation results for binary neurons in **(C)** is due to different realizations of the random connectivity. **(E,F)** are the same as **(A,B)** but for the presence of a synaptic delay *d* = 10 ms instead of *d* = 0.1 ms.

The two alternative methods of linearization of ϕ are illustrated in Figure 5. In the given example, the linearization procedure taking into account the fluctuations of the input signal results in a smaller effective slope 〈ϕ′〉 than taking the slope ϕ′(*a*) at the mean activity *a* near its maximum. Averaging the slope 〈ϕ′〉 over this distribution fits simulation results better than ϕ′(*a*) calculated at the mean of *a*, as shown in Figure 6.

The finite slope of the non-linear gain function can be understood as resulting from the combination of a hard threshold with an intrinsic local source of noise. The inverse strength of this noise determines the slope parameter β (Ginzburg and Sompolinsky, 1994). In this sense, the network model contains two sources of noise, the explicit local noise, quantified by β and the fluctuating synaptic input interpreted as self-generated noise on the network level, quantified by σ. Even in the absence of local noise (β → ∞), the above mentioned linearization is applicable and yields a finite effective slope 〈ϕ′〉 (27). In the latter case the resulting effective synaptic weight is independent of the original synapse strength (Grytskyy et al., 2013).

We now extend the classical treatment of covariances in binary networks (Ginzburg and Sompolinsky, 1994) by synaptic conduction delays. In (25) *F*_*i*_(**n**, *t*) must therefore be understood as a functional acting on the function **n**(*t*′) for *t*′ ∈ [−∞, *t*], so that also synaptic connections with time delay *d* can be realized. We define an effective weight vector to absorb the gain factor as **w**_*i*_ = β_*i*_**J**_*i*_, with either β_*i*_ = ϕ′(μ) or β_*i*_ = 〈ϕ′〉 depending on the linearization procedure, and expand the right hand side of (24) to obtain

$$\langle F_{i}(n,t)(n_{j}(s)-a_{j}(s))\rangle =\sum_{k=1}^{N}w_{ik}c_{kj}(t-d,s).$$

Thus the cross-covariance fulfills the matrix delay differential equation

(28) $$\tau \frac{d}{dt}c(t,s)+c(t,s)=wc(t-d,s).$$

This differential equation is valid for *t* > *s*. For the stationary solution, the differential equation only depends on the relative timing *u* = *t* − *s*

(29) $$\tau \frac{d}{du}c(u)+c(u)=wc(u-d).$$

The same linearization applied to (23) results in the boundary condition for the solution of the previous equation

(30) $$2c(0)=wc(-d)+{\left(wc(-d)\right)}^{T}$$

or, if we split **c** into its diagonal and its off-diagonal parts **c**_*a*_ and **c**_≠_

(31) $$\begin{array}{c} \begin{array}{l} 2c_{\neq }(0)=wc_{\neq }(-d)+{\left(wc_{\neq }\left(-d\right)\right)}^{T}+O\\ \text{ with }O=wc_{a}(-d)+{\left(wc_{a}\left(-d\right)\right)}^{T}. \end{array} \end{array}$$

In the following section we use this representation to demonstrate the equivalence of the covariance structure of binary networks to the solution for OUP with input noise.

### 3.1. Equivalence of binary neurons and ornstein–uhlenbeck processes

In the following subsection we show that the same Equations (29) and (31) for binary neurons also hold for the Ornstein-Uhlenbeck process (OUP) with input noise. In doing so here we also extend the existing framework of OUP (Risken, 1996) to synaptic conduction delays *d*. A network of such processes is described by

(32) $$\tau \frac{d}{dt}r(t)+r(t)=wr(t-d)+x(t),$$

where **x** is a vector of pairwise uncorrelated white noise with 〈**x**(*t*)〉_*x*_ = 0 and 〈*x*_*i*_(*t*)*x*_*j*_(*t* + *t*′)〉_*x*_ = δ_*ij*_δ(*t*′)ρ^2^. With the help of the Green's function *G* satisfying $\left(\tau \frac{d}{dt}+1)G(t)=\delta (t\right)$, namely $G(t)=\frac{1}{\tau }\theta (t)e^{-t/\tau }$, we obtain the solution of Equation (32) as

$$r(t)=\tau G(t)r(0)+\int_{0}^{t}G(t-t^{\prime })(wr(t^{\prime }-d)+x(t^{\prime }))dt^{\prime }.$$

The equation for the fluctuations δ**r**(*t*) = **r**(*t*) − 〈**r**(*t*)〉_*x*_ around the expectation value

$$\delta r(t)=\int_{0}^{t}G(t-t^{\prime })(w\delta r(t^{\prime }-d)+x(t^{\prime }))dt^{\prime }$$

coincides with the noisy rate model with input noise (4) with delay *d* and convolution kernel *h* = *G*. In the next step we investigate the covariance matrix *c*_*ij*_(*t, s*) = 〈δ*r*_*i*_(*t* + *s*)δ*r*_*j*_(*t*)〉_*x*_ to show for which choice of parameters the covariance matrices for the binary model and the OUP with input noise coincide. To this end we derive the differential equation with respect to the time lag *s* for positive lags *s* > 0

(33) $$\begin{array}{c} \begin{array}{l} \tau \frac{d}{ds}c(t,s)={\langle \tau \frac{d}{ds}\delta r(t+s)\delta r^{T}(t)\rangle }_{x}\\ \;={\langle \left(w\delta r(t+s-d)-\delta r(t+s)+x(t+s))\delta r^{T}(t\right)\rangle }_{x}\\ \;=wc(t,s-d)-c(t,s), \end{array} \end{array}$$

where we used 〈**x**(*t* + *s*))δ**r**(*t*)〉_*x*_ = 0, because the noise is realized independently for each time step and the system is causal. Equation (33) is identical to the differential equation satisfied by the covariance matrix (28) for binary neurons (Ginzburg and Sompolinsky, 1994). To determine the initial condition of (33) we need to take the limit **c**(*t*, 0) = lim_*s* → +0_**c**(*t, s*). This initial condition can be obtained as the stationary solution of the following differential equation

$$\begin{array}{l} \tau \frac{d}{dt}c(t,0)=\underset{s\to +0}{\lim}\left({\langle \tau \frac{d}{dt}\delta r(t+s)\delta r^{T}(t)\rangle }_{x}+{\langle \delta r(t+s)\tau \frac{d}{dt}\delta r^{T}(t)\rangle }_{x}\right)\\ \;=\underset{s\to +0}{\lim}({\langle (w\delta r(t+s-d)-\delta r(t+s)+x(t+s))\delta r^{T}(t)\rangle }_{x}\\ \;+{\langle \delta r(t+s)(\delta r^{T}(t-d)w^{T}-\delta r^{T}(t)+x^{T}(t))\rangle }_{x})\\ \;=-2c(t,0)+wc(t,-d)+c(t-d,d)w^{T}+D. \end{array}$$

Here we used that 〈**x**(*t* + *s*)δ**r**^*T*^(*t*)〉 vanishes due to independent noise realizations and causality and

$$\begin{array}{l} D=\underset{s\to +0}{\lim}{\langle \delta r(t+s)x^{T}(t)\rangle }_{x}\\ \;=\underset{s\to +0,\;s<d}{\lim}\int_{0}^{t+s}G(t+s-t^{\prime })(w\underset{=0\text{ causality}}{\underset{︸}{{\langle \delta r(t^{\prime }-d)x^{T}(t)\rangle }_{x}}}+\underset{=1\delta (t-t^{\prime })\rho^{2}}{\underset{︸}{{\langle x(t^{\prime })x^{T}(t)\rangle }_{x}}})dt^{\prime }\\ \;=\underset{s\to +0,\;s<d}{\lim}\int_{0}^{t+s}G(t+s-t^{\prime })1\delta (t-t^{\prime })\rho^{2}dt^{\prime }\\ \;=\underset{s\to +0,\;s<d}{\lim}G(s)1\rho^{2}=\frac{1}{\tau }1\rho^{2}. \end{array}$$

In the stationary state, **c** only depends on the time lag *s* and is independent of the first time argument *t*, which, with the symmetry **c**(−*d*)^*T*^ = **c**(*d*) yields the additional condition for the solution of (33)

$$2c(0)=wc(-d)+{\left(wc(-d)\right)}^{T}+D$$

or, if **c** is split in diagonal and off-diagonal parts **c**_*a*_ and **c**_≠_, respectively,

$$\begin{array}{l} 2c_{\neq }(0)=wc_{\neq }(-d)+{\left(wc_{\neq }\left(-d\right)\right)}^{T}+O\\ 2c_{a}(0)=wc_{\neq }(-d)+{\left(wc_{\neq }\left(-d\right)\right)}^{T}+D \end{array}$$

with **O** = **wc**_*a*_(−*d*) + (**wc**_*a*_(−*d*))^*T*^. In the equation for the autocovariance **c**_*a*_ the first two terms are contributions due to the cross covariance. In the state of asynchronous network activity with *c*_*ij*_ ~ *N*^−1^ for *i*≠ *j* these terms are typically negligible in comparison to the third term because ∑_*k*_ *w*_*ik*_*c*_*ki*_ ~ *wKN*^−1^ = *pw*, which is typically smaller than 1 for small effective weights *w* < 1 and small connection probabilities *p* « 1. In this approximation with (33) the temporal shape of the autocovariance function is exponentially decaying with time constant τ. With **c**_*a*_(0) ≈ **D**/2 the approximate solution for the autocovariance is

(34) $$c_{a}(t)=\frac{D}{2}\exp\left(-\frac{\left|t\right|}{\tau }\right)\text{​}.$$

The cross covariance then satisfies the initial condition

$$\begin{array}{l} 2c_{\neq }(0)=wc_{\neq }(-d)+{\left(wc_{\neq }\left(-d\right)\right)}^{T}+O\\ \;O=wD/2+{\left(wD/2\right)}^{T}, \end{array}$$

which coincides with (31) for binary neurons if the diagonal matrix containing the zero time autocorrelations **c**_*a*_(0) for binary neurons is equal to **D**/2, i.e., if the amplitude of the input noise ρ^2^ = 2τ *a*(1 − *a*) and the effective linear coupling satisfies **w**_*i*_ = β_*i*_**J**_*i*_. Figure 6 shows simulation results for population averaged covariance functions in binary networks and in networks of OUPs with input noise where the parameters of the OUP network are chosen according to the requirements derived above. The theoretical results (18) agree well with the direct simulations of both systems. For comparison, both methods of linearization, as explained above, are shown. The linearization procedure which takes into account the noise on the input side of the non-linear gain function results in a more accurate prediction. Moreover, the results derived here extend the classical theory (Ginzburg and Sompolinsky, 1994) by considering synaptic conduction delays. Figure 8 shows the decomposition of the covariance structure for a non-zero delay *d* = 3 ms. For details of the implementation see “Implementation of binary neurons in a spiking simulator code”. The explicit effect of introducing delays into the system, such as the appearance of oscillations in the time dependent covariance, is presented in **(E,F)** of Figure 6, differing from **(A,B)** of this figure, respectively, only in the delay (*d* = 10 ms for **(E,F)**, *d* = 0.1 ms for **(A,B)**).

## 4. Hawkes processes

In the following section we show that to linear order the covariance functions in networks of Hawkes processes (Hawkes, 1971) are equivalent to those in the linear rate network with output noise. Hawkes processes generate spikes randomly with a time density given by **r**(*t*), where neuron *i* generates spikes at a rate *r*_*i*_(*t*), realized independently within each infinitesimal time step. Arriving spike trains **s** influence **r** according to

(35) $$r(t)=\nu +(h_{d}*Js)(t),$$

with the connectivity matrix **J** and the kernel function *h*_*d*_ including the delay. Here ν is a constant base rate of spike emission assumed to be equal for each neuron. Here we employ the implementation of the Hawkes model in the NEST simulator (Gewaltig and Diesmann, 2007). The implementation is described in “Implementation of Hawkes neurons in a spiking simulator code”.

Given neuron *j* spiked at time *u* ≤ *t*, the probability of a spike in the interval [*t, t*+δ*t*) for neuron *i* is 1 if *i* = *j*, *u* = *t* (the neuron spikes synchronously with itself) and *r*_*i*_(*t*)δ*t* + *o*(δ*t*^2^) otherwise. Considering the system in the stationary state with the time averaged activity **r** = 〈**s**(*t*)〉 we obtain a convolution equation for time lags τ ≥ 0 for the covariance matrix with the entry *c*_*ij*_(τ) for the covariance between spike trains of neurons *i* and *j*

(36) $$\begin{array}{l} c(\tau )=\langle s(t+\tau )s^{T}(t)\rangle -\langle s(t+\tau )\rangle \langle s^{T}(t)\rangle \\ \;=\langle (\delta (\tau )1+r(t+\tau ))s^{T}(t)\rangle -\bar{r}{\bar{r}}^{T}\\ \;=\langle r(t+\tau )(s^{T}(t)-{\bar{r}}^{T})\rangle +D_{\bar{r}}\\ \;=\langle (\nu +(h_{d}*Js)(t+\tau ))(s^{T}(t)-{\bar{r}}^{T})\rangle +D_{\bar{r}}\\ \;=h_{d}*J\langle s(t+\tau )(s^{T}(t)-{\bar{r}}^{T})\rangle +D_{\bar{r}}\\ \;=(h_{d}*Jc)(\tau )+D_{\bar{r}}, \end{array}$$

with the diagonal matrix **D**_**r**_ = δ(τ)diag(**r**), which has been derived earlier (Hawkes, 1971). If the rates of all neurons are equal, **r**_*i*_ = *r*, all entries in the diagonal matrix are the same, **D**_*r*_ = δ(τ)**1***r*. In the subsequent section we demonstrate that the same convolution Equation (36) holds for the linear rate with output noise.

### 4.1. Convolution equation for linear noisy rate neurons

For the linear rate model with output noise we use Equation (3) for time lags τ > 0 to obtain a convolution equation for the covariance matrix of the output signal vector **y** = **r** + **x** as

(37) $$\begin{array}{l} c(\tau )=\langle y(t+\tau )(y^{T}(t)-{\bar{r}}^{T})\rangle \\ \;=\langle (h_{d}*wy+x)(t+\tau )(y^{T}(t)-{\bar{r}}^{T})\rangle \\ \;=(h_{d}*wc)(\tau )+\langle x(t+\tau )(r^{T}(t)-{\bar{r}}^{T})\rangle +\langle x(t+\tau )x^{T}(t)\rangle \\ \;=(h_{d}*wc)(\tau )+D, \end{array}$$

where we utilized that due to causality the random noise signal generated at *t*+τ has no influence on **r**(*t*), so the respective correlation vanishes. **D** is the covariance of the noise as in (11), *D*_*ij*_(τ) = 〈*x*_*i*_(*t*)*x*_*j*_(*t* + τ)〉 = δ_*ij*_δ(τ)ρ^2^. If ρ is chosen such that ρ^2^ coincides with the averaged activity *r* in a network of Hawkes neurons and the connection matrix **w** is identical to **J** of the Hawkes network, the Equations (36) and (37) are identical. Therefore the cross spectrum of both systems is given by (11).

### 4.2. Non-linear self-consistent rate in rectifying hawkes networks

The convolution Equation (36) for the covariance matrix of Hawkes neurons is exact if no element of **r** is negative, which is particularly the case for a network of only excitatory neurons. Especially in networks including inhibitory couplings, the intensity *r*_*i*_ of neuron *i* may assume negative values. A neuron with *r*_*i*_ < 0 does not emit spikes, so the instantaneous rate is given by λ_*i*_ = [*r*_*i*_(*t*)]_+_ = θ(*r*_*i*_(*t*)) *r*_*i*_(*t*), with the Heaviside function θ. We now take into account this effective nonlinearity –the rectification of the Hawkes model neuron– in a similar manner as we already used to linearize binary neurons. If the network is in the regime of low spike rates, the fluctuations in the input of each neuron due to the Poissonian arrival of spikes are large compared to the fluctuations due to the time varying intensities **r**(*t*). Considering the same homogeneous network structure as described in “Population-averaged covariances,” the input statistics is identical for each cell *i*, so the mean activity λ_0_ = 〈λ_*i*_〉 is the same for all neurons *i*. The superposition of the synaptic inputs to neuron *i* cause an instantaneous intensity *r*_*i*_ that follows approximately a Gaussian distribution 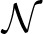
(μ,σ,*r*_*i*_) with mean μ = 〈*r*〉 = ν+λ_0_*KJ*(1 − *g*γ) and standard deviation $\sigma =\sqrt{\langle r^{2}\rangle -{\langle r\rangle }^{2}}=J\sqrt{\frac{\lambda_{0}}{2\tau }K(1+g^{2}\gamma )}$. These expressions hold for the exponential kernel (5) due to Campbell's theorem (Papoulis and Pillai, 2002), because of the stochastic Poisson-like arrival of incoming spikes, where the standard deviation of the spike count is proportional to the square root of the intensity λ_0_. The rate λ_0_ is accessible by explicit integration over the Gaussian probability density as 
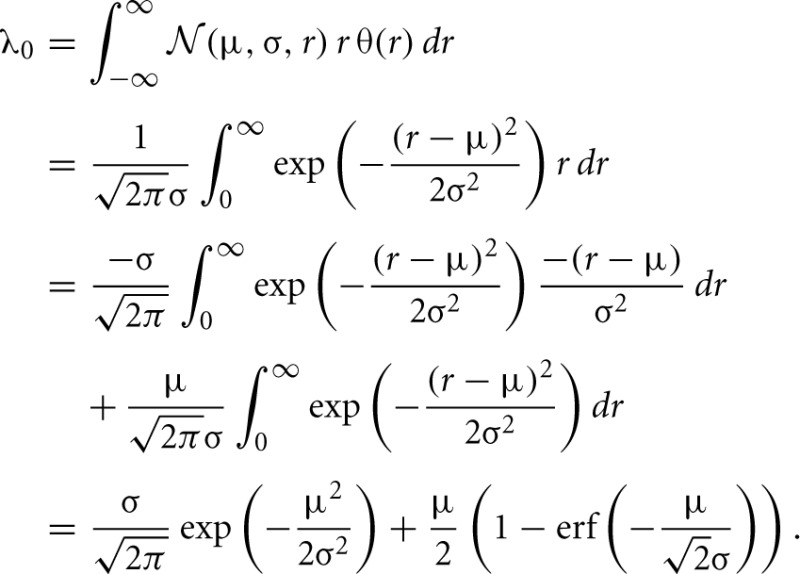


This equation needs to be solved self-consistently (numerically or graphically) to determine the rate in the network, as the right hand side depends on the rate λ_0_ itself through μ and σ. Rewritten as

(38) $$\begin{array}{l} \;\lambda_{0}=\frac{\sigma }{\sqrt{2\pi }}\exp\left(-\frac{\mu^{2}}{2\sigma^{2}}\right)+\mu P_{\mu ,\sigma }(r>0)\\ P_{\mu ,\sigma }(r>0)=\frac{1}{2}-\frac{1}{2}\text{erf}\left(-\frac{\mu }{\sqrt{2}\sigma }\right), \end{array}$$

*P*_μ,σ_(*r* > 0) is the probability that the intensity of a neuron is above threshold and therefore contributes to the transmission of a small fluctuation in the input. A neuron for which *r* < 0 acts as if it was absent. Hence we can treat the network with rectifying neurons completely analogous to the case of linear Hawkes processes, but multiply the synaptic weight *J* or −*gJ* of each neuron with *P*_μ,σ_(*r* > 0), i.e., the linearized connectivity matrix is

(39) $$w=P_{\mu ,\sigma }(r>0)J.$$

**Figure 7:**
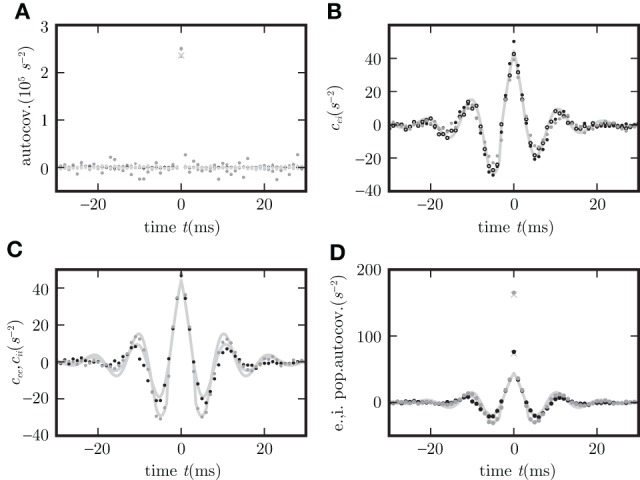
**Covariance structure in spiking networks corresponds to OUP with output noise. (A)** Autocovariance obtained by direct simulation of the LIF (black), Hawkes (gray), and OUP (light gray) models for excitatory (dots) and inhibitory neurons (crosses). **(B)** Covariance *c*_
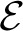

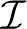
_ averaged over disjoint pairs of neurons for LIF (black dots), Hawkes (gray dots), and OUP with output noise (empty circles). **(C)** Covariance averaged over disjoint pairs of neurons of the same type. **(D)** Autocovariance of the population averaged activity. Averages in **(C,D)** over excitatory neurons as black dots, over inhibitory neurons as gray dots. Corresponding theoretical predictions (21) are plotted as light gray curves in all panels except **(A)**. Light gray diagonal crosses in **(A,D)** denote theoretical peak positions determined by the firing rate *r* as *r*Δ*t* (where Δ*t* = 0.1 ms is the time resolution of the histogram).

Figure 7 shows the agreement of the covariance functions obtained from direct simulation of the network of Hawkes processes and the analytical solution (21) with average firing rate λ_0_ determined by (38), setting the effective strength of the noise ρ^2 = λ_0_^, and the linearized coupling as described above. The detailed procedure for choosing the parameters in the direct simulation is described together with the implementation of the Hawkes model in “Implementation of Hawkes neurons in a spiking simulator code”.

## 5. Leaky integrate-and-fire neurons

In this section we consider a network of LIF model neurons with exponentially decaying postsynaptic currents and show its equivalence to the network of OUP with output noise, valid in the asynchronous irregular regime. A spike sent by neuron *j* at time *t* arrives at the target neuron *i* after the synaptic delay *d*, elicits a synaptic current *I*_*i*_ that decays with time constant τ_*s*_ and causes a response in the membrane potential *V*_*i*_ proportional to the synaptic efficacy *J*_*ij*_. With the time constant τ_*m*_ of the membrane potential, the coupled set of differential equations governing the subthreshold dynamics of a single neuron *i* is (Fourcaud and Brunel, 2002)

(40) $$\begin{array}{l} \tau_{m}\frac{dV_{i}}{dt}=-V_{i}+I_{i}(t)\\ \;\tau_{s}\frac{dI_{i}}{dt}=-I_{i}+\tau_{m}\sum_{j=1,j}^{N}J_{ij}s_{j}(t-d), \end{array}$$

where the membrane resistance was absorbed into the definitions of *J*_*ij*_ and *I*_*i*_. If *V*_*i*_ reaches the threshold *V*_θ_ at time point *t*^*i*^_*k*_ the neuron emits an action potential and the membrane potential is reset to *V*_*r*_, where it is clamped for the refractory time τ_*r*_. The spiking activity of neuron *i* is described by this sequence of action potentials, the spike train *s*_*i*_(*t*) = ∑_*k*_δ(*t* − *t*^*i*^_*k*_). The dynamics of a single neuron is deterministic, but in network states of asynchronous, irregular activity and in the presence of external Poisson inputs to the network, the summed input to each cell can well be approximated as white noise (Brunel, 2000) with first moment μ_*i*_ = τ_*m*_∑_*j*_*J*_*ij*_*r*_*j*_ and second moment σ^2^_*i*_ = τ_*m*_∑_*j*_*J*^2^_*ij*_*r*_*j*_, where *r*_*j*_ is the stationary firing rate of neuron *j*. The stationary firing rate of neuron *i* is then given by Fourcaud and Brunel (2002)

(41) $$\begin{array}{l} \;r_{i}^{-1}=\tau_{r}+\tau_{m}\sqrt{\pi }\left(F(y_{\theta })-F(y_{r})\right)\\ \;f(y)=e^{y^{2}}(1+\text{erf}(y))\;F(y)=\int^{y}f(y)dy\\ \text{with }y_{\theta ,r}=\frac{V_{\theta ,r}-\mu_{i}}{\sigma_{i}}+\frac{\alpha }{2}\sqrt{\frac{\tau_{s}}{\tau_{m}}}\;\alpha =\sqrt{2}\left|\zeta \left(\frac{1}{2}\right)\right|, \end{array}$$

with Riemann's zeta function ζ. The response of the LIF neuron to the injection of an additional spike into afferent *j* determines the impulse response *w*_*ij*_*h*(*t*) of the system. The time integral *w*_*ij*_ = *w*_*ij*_ ∫^∞^_0_*h*(*t*)*dt* is the DC-susceptibility, which can formally be written as the derivative of the stationary firing rate by the rate of the afferent *r*_*j*_, which, evaluated by help of (41), yields (Helias et al., 2013, Results and App. A)

(42) $$\begin{array}{c} \begin{array}{l} \;w_{ij}=\frac{\partial r_{i}}{\partial r_{j}}=\alpha J_{ij}+\beta J_{ij}^{2}\\ \text{with }\alpha =\sqrt{\pi }{\left(\tau_{m}r_{i}\right)}^{2}\frac{1}{\sigma_{i}}\left(f(y_{\theta })-f(y_{r})\right)\\ \text{ and }\beta =\sqrt{\pi }{\left(\tau_{m}r_{i}\right)}^{2}\frac{1}{2\sigma_{i}^{2}}\left(f(y_{\theta })\frac{V_{\theta }-\mu_{i}}{\sigma_{i}}-f(y_{r})\frac{V_{r}-\mu_{i}}{\sigma_{i}}\right). \end{array} \end{array}$$

In the strongly fluctuation-driven regime, the temporal behavior of the kernel *h* is dominated by a single exponential decay, whose time constant can be determined empirically. In a homogeneous random network the firing rates of all neurons are identical *r*_*i*_ = *r* and follow from the numerical solution of the self-consistency Equation (41). Approximating the autocovariance function of a single spike train by a δ-peak scaled by the rate *r*δ(*t*), one obtains for the covariance function **c** between pairs of spike trains the same convolution Equation (36) as for Hawkes neurons (Helias et al., 2013, cf. equation 5). As shown in “Convolution equation for linear noisy rate neurons” this convolution equation coincides with that of a linear rate model with output noise (37), where the diagonal elements of **D** are chosen to agree to the average spike rate ρ^2^ = *r*. The good agreement of the analytical cross covariance functions (21) for the OUP with output noise and direct simulation results for LIF are shown in Figure 7.

## 6. Discussion

In this work we describe the path to a unified theoretical view on pairwise correlations in recurrent networks. We consider binary neuron models, LIF models, and linear point process models. These models containing a non-linearity (spiking threshold in spiking models, non-linear sigmoidal gain function in binary neurons, strictly positive rates in Hawkes processes) are linearized, taking into account the distribution of the fluctuating input.

The work presents results for several neuron models: We derive analytical expressions for delay-coupled OUP with input and with output noise, we extend the analytical treatment for stochastic binary neurons to the presence of synaptic delays, present a method that takes into account network-generated noise to determine the effective gain function, extend the theory of Hawkes processes to the existence of delays and inhibition, and present in Equation (12) a condition for the onset of global oscillations caused by delayed feedback, generalized to feedback pathways through different eigenvalues of the connectivity.

Some results qualitatively extend the existing theory (delays, inhibition), others improve the accuracy of existing theories (linearization including fluctuations). More importantly, our approach enables us to demonstrate the equivalence of each of these models after linear approximation to a linear model with fluctuating continuous variables. The fact that linear perturbation theory leads to effective linear equations is of course not surprising, but the analytical procedure firstly enables a mapping between models that conserves quantitative results and secondly allows us to uncover common structures underlying the emergence of correlated activity in recurrent networks. For the commonly appearing exponentially decaying response kernel function, these rate models coincide with the OUP (OUP, Uhlenbeck and Ornstein, 1930; Risken, 1996). We find that the considered models form two groups, which, in linear approximation merely differ by a matrix valued factor scaling the noise and in the choice of variables interpreted as neural activity. The difference between these two groups corresponds to the location of the noise: spiking models—LIF models and Hawkes models—belong to the class with noise on the output side, added to the activity of each neuron. The non-spiking binary neuron model corresponds to an OUP where the noise is added on the input side of each neuron. The closed solution for the correlation structure of OUP holds for both classes.

**Figure 8:**
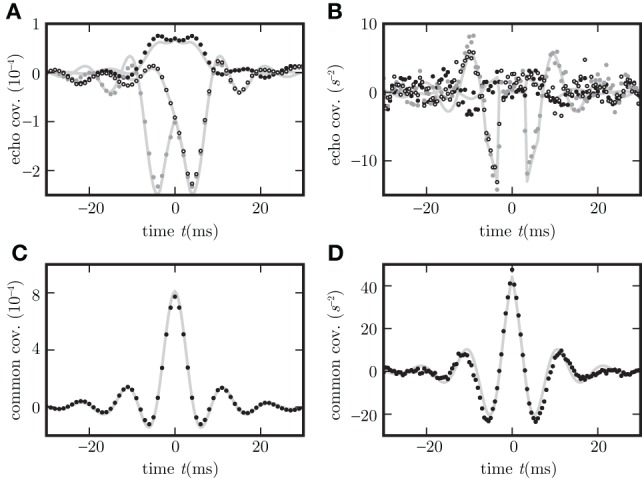
**Different echo terms for spiking and non-spiking neurons**. Binary non-spiking neurons shown in **(A,C)** and LIF in **(B,D)**. **(A)**,**(B)** Echo terms by direct influence of the neuron's output on the network in dependence of neuron types (in **A,B** *c*_
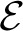

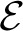
_,*c*_
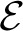

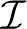
_, and *c*_
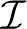

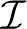
_ are plotted as black, gray dots and circles). **(C,D)**, Contributions to the covariance evoked by correlated and common input (black dots) measured with help of auxiliary model neurons which do not provide feedback to the network. Corresponding theoretical predictions (16) are plotted as light gray curves throughout.

We identify different contributions to correlations in recurrent networks: the solution for output noise is split into three terms corresponding to the δ-peak in the autocovariance, the covariance caused by shared input, and the direct synaptic influence of stochastic fluctuations of one neuron on another–the latter echo terms are equal to propagators acting with delays (Helias et al., 2013). A similar splitting into echo and correlated input terms for the case of input noise is shown in Figure 8. For increasing network size *N* → ∞, keeping the connection probability *p* fixed, so that *K* = *pN*, and with rescaled synaptic amplitudes *J* ~ 1/$\sqrt{N}$ (van Vreeswijk and Sompolinsky, 1996; Renart et al., 2010) the echo terms vanish fastest. Formally this can be seen from (18): the multiplicative factor of the common covariance term φ_4_ does not change with *N* while the other coefficients decrease. So ultimately all four entries of the matrix **c** have the same time dependence determined by the common covariance term φ_4_. In particular the covariance between excitation and inhibition *c*_
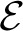

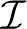
_ becomes symmetric in this limit. This finally provides a quantitative explanation of the observation made in (Renart et al., 2010) that the time-lag between excitation and inhibition vanishes in the limit of infinitely large networks. For a different synaptic rescaling *J* ~ *N*^−1^ while keeping ρ^2^ constant by appropriate additional input to each neuron (see Helias et al., 2013 applied to the LIF model), all multiplicative factors decrease ~ *N*^−1^ and so does the amplitude of all covariances. Hence the asymmetry of *c*_
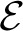

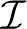
_ does not vanish in this limit. The same results hold for the case of output noise where the term with φ_1_ describes the common input part of the covariance. In this case and for finite network size, *c*_
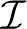

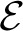
_ coincides with *c*_
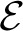

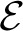
_ and *c*_
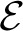

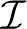
_ with *c*_
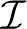

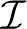
_ for *t* > 0, having a discontinuous jump at the time of the synaptic delay *t* = *d*. For time lags smaller than the delay all four covariances coincide. This is due to causality, as the second neuron cannot feel the influence of a fluctuation that happened in the first neuron less than one synaptic delay before. The covariance functions for systems corresponding to an OUP with input noise contain neither discontinuities nor sharp peaks at *t* = *d*, but *c*_
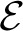

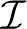
_ and *c*_
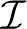

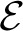
_ have maxima and minima near this location. This observation can be interpreted as a result of the stochastic nature of the binary model where changes in the input influence the state of the neuron only with a certain probability. So, the entries of **c** in this case take different values for |*t*| < *d* but show the tendency to approach each other with increasing |*t*| » *d*. This tendency increases with network size. Our analytical solutions (18) for input noise and (21) for output noise hence explain the model-class dependent differences in the shape of covariance functions.

The two above mentioned synaptic scaling procedures are commonly termed “strong coupling” (*J* ~ 1/$\sqrt{N}$) and “weak coupling” (*J* ~ 1/*N*), respectively. The results shown in Figure 6 were obtained for *J* = 2/$\sqrt{N}$ and β = 0.5, so the number of synapses required to cause a notable effect on the gain function is 1/(β *J*) = $\sqrt{N}$, which is small compared to the number of incoming synapses *pN*. Hence the network is in the strong coupling regime. Also note that for infinite slope of the gain function, β → ∞, the magnitude of the covariance becomes independent of the synaptic amplitude *J*, in agreement with the linear theory presented here. This finding can readily be understood by the linearization procedure, presented in the current work, that takes into account the network- generated fluctuations of the total input. The amplitude σ of these fluctuations scales linearly in *J* and the effective susceptibility depends on *J*/σ in the case β → ∞, explaining the invariance (Grytskyy et al., 2013). In the current manuscript we generalized this procedure to finite slopes β and to other models than the binary neuron model.

Our approach enables us to map results obtained for one neuron model to another, in particular we extend the theory of all considered models to capture synaptic conduction delays, and devise a simpler way to obtain solutions for systems considered earlier (Ginzburg and Sompolinsky, 1994). Our derivation of covariances in spiking networks does not rely on the advanced Wiener-Hopf method (Hazewinkel, 2002), as earlier derivations (Hawkes, 1971; Helias et al., 2013) do, but only employs elementary methods. Our results are applicable for general connectivity matrices, and for the purpose of comparison with simulations we explicitly derive population averaged results. The averages of the dynamics of the linear rate model equations are exact for random network architectures with fixed out-degree, and approximate for fixed in-degree. Still, for non-linear models the linearization for fixed in-degree networks are simpler, because the homogeneous input statistics results in an identical linear response kernel for all cells. Finally we show that the oscillatory properties of networks of integrate-and-fire models (Brunel, 2000; Helias et al., 2013) are model-invariant features of all of the studied dynamics, given inhibition acts with a synaptic delay. We relate the collective oscillations to the pole structure of the cross spectrum, which also determines the power spectra of population signals such as EEG, ECoG, and the LFP.

The presented results provide a further step to understand the shape and to unify the description of correlations in recurrent networks. We hope that our analytical results will be useful to constrain the inverse problem of determining the synaptic connectivity given the correlation structure of neurophysiological activity measurements. Moreover the explicit expressions for covariance functions in the time domain are a necessary prerequisite to understand the evolution of synaptic amplitudes in systems with spike-timing dependent plasticity and extend the existing methods (Burkitt et al., 2007; Gilson et al., 2009, 2010) to networks including inhibitory neurons and synaptic conduction delays.

### Conflict of interest statement

The authors declare that the research was conducted in the absence of any commercial or financial relationships that could be construed as a potential conflict of interest.

## Figures and Tables

**Algorithm 1:**
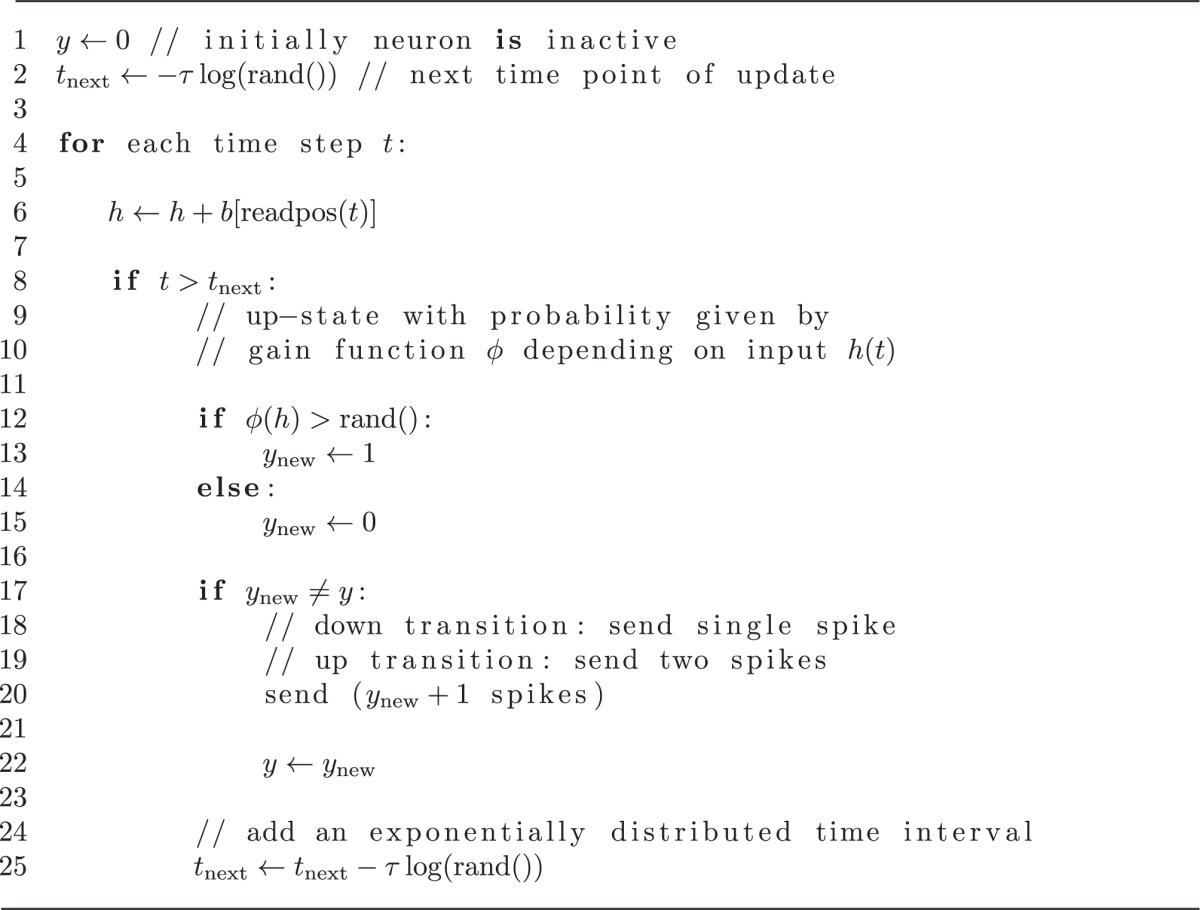
**Update function of a binary neuron embedded in the spiking network simulator NEST**.

*The function* readpos(t) *returns a position in the ring buffer b corresponding to the current time point.*

**Algorithm 2:**
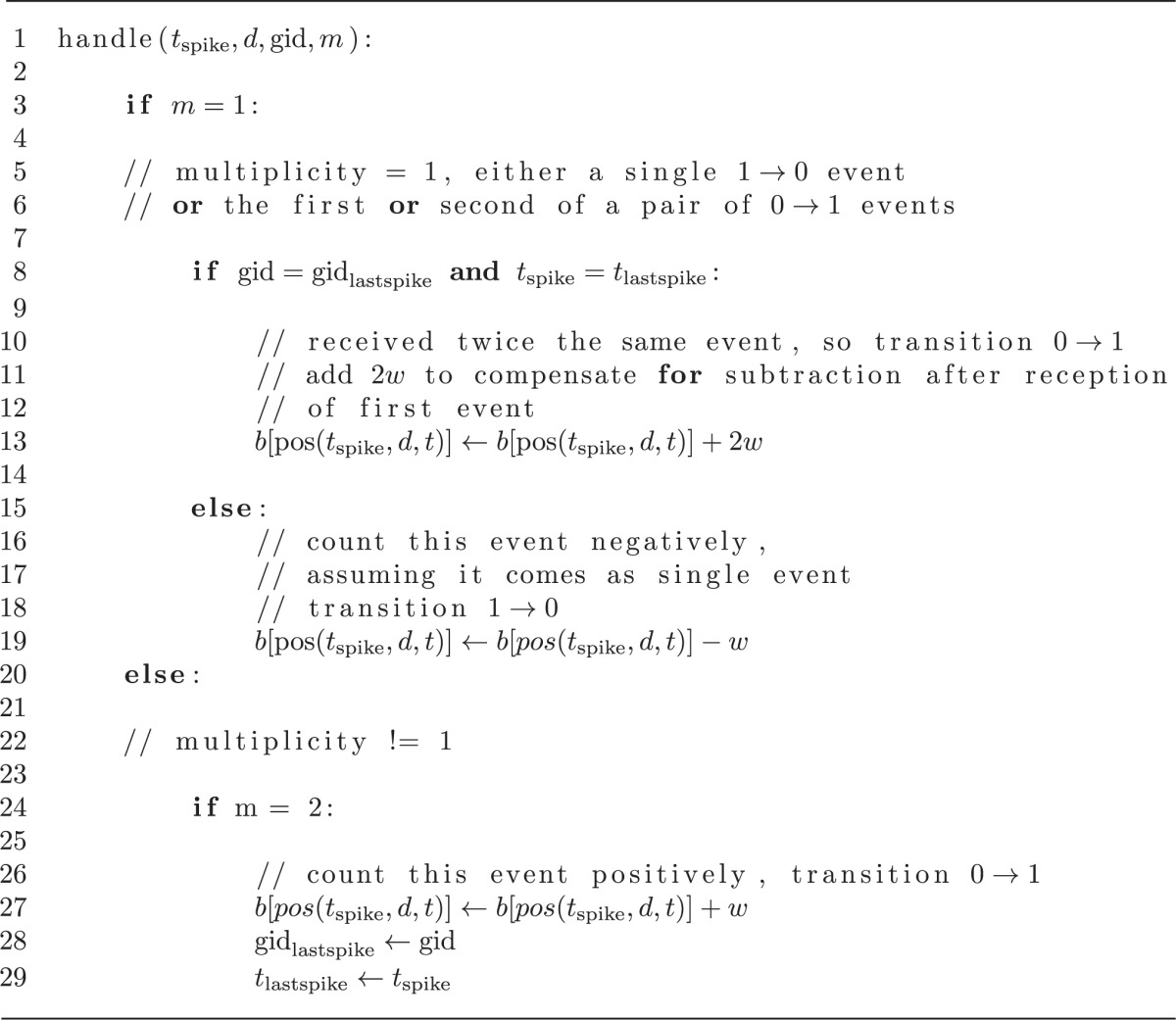
**Input spike handler of a binary neuron embedded in the spiking network simulator NEST**.

*The simulation kernel calls the handle function for each spike event to be delivered to the neuron. A spike event is characterized by the time point of occurrence t*_spike_*, the synaptic delay d after which the event should reach the target, the global id* gid *identifying the sending neuron, and the multiplicity m ≥ 1, indicating the reception of multiple spike events. The function* pos*(t*_spike_, *d, t) returns the position in the ring buffer b to which the spike is added so that it will be read at time t + d by the update function of the neuron, see Alg. 1.*

## Appendix

### Calculation of the population averaged cross covariance in time domain

We obtain the population averaged cross spectrum for the Ornstein-Uhlenbeck process with input noise by inserting the averaged connectivity matrix **w** = **M** (14) into (8). The two eigenvalues of **M** are 0 and *L* = *Kw*(1 − γ*g*). Taking these into account, we first rewrite the term

$$\begin{array}{l} {\left(H_{d}{\left(\omega \right)}^{-1}-M\right)}^{-1}\\ \;=\det{\left(H_{d}{\left(\omega \right)}^{-1}-M\right)}^{-1}\left(\begin{array}{c} \begin{array}{cc} H_{d}{\left(\omega \right)}^{-1}+Kw\gamma g & -Kw\gamma g\\ Kw & H_{d}{\left(\omega \right)}^{-1}-Kw \end{array} \end{array}\right)\\ \;={\left(\left(H_{d}{\left(\omega \right)}^{-1}-0)(H_{d}{\left(\omega \right)}^{-1}-L\right)\right)}^{-1}\left(H_{d}{\left(\omega \right)}^{-1}1+Kw\left(\begin{array}{c} \begin{array}{cc} \gamma g & -\gamma g\\ 1 & -1 \end{array} \end{array}\right)\right)\\ \;=f(\omega )\left(1+Kw\left(\begin{array}{c} \begin{array}{cc} \gamma g & -\gamma g\\ 1 & -1 \end{array} \end{array}\right)H_{d}(\omega )\right), \end{array}$$

where we introduced *f*(ω) = (*H*_*d*_(ω)^−1^ − *L*)^−1^. The corresponding transposed and conjugate complex term follows analogously. Hence we obtain the expression for the cross spectrum (17). The residue of *f*(ω) at ω = *z*_*k*_(*L*) is

$$\begin{array}{l} \text{ Res}(f,\omega =z_{k}(L))=\underset{\omega_{1}\to \omega }{\lim}\frac{\omega_{1}-\omega }{f^{-1}(\omega_{1})}\\ \overset{{\text{l}}^{\prime }\text{Hopital}}{=}\underset{\omega_{1}\to \omega }{\lim}\frac{1}{{\left(f^{-1}\right)}^{\prime }(\omega_{1})}={\left(\frac{d(e^{i\omega d}(1+i\omega \tau ))}{d\omega }\right)}^{-1}\\ \;={\left(ide^{i\omega d}(1+i\omega \tau )+i\tau e^{i\omega d}\right)}^{-1}\\ \;={\left(idL+i\tau e^{i\omega d}\right)}^{-1}, \end{array}$$

where in the last step we used the condition for a pole *H*_*d*_(*z*_*k*_)^−1^ = *e*^*iz*_*k*_*d*^(1 + *iz*_*k*_τ) = *L* (see “Spectrum of the dynamics”). The residue of *H*_*d*_(ω) at $z(0)=\frac{i}{\tau }$ is $-\frac{i}{\tau }e^{d/\tau }$. Using the residue theorem, we need to sum over all poles within the integration contour $\left\{z_{k}(L)|k\in \mathbb{N}\right\}\cup \frac{i}{\tau }$ to get the expression for $c(t)=\frac{1}{2\pi }\int_{-\infty }^{+\infty }C(\omega )e^{i\omega t}d\omega =i\sum_{z\in \left\{z_{k}(L)|k\in \mathbb{N}\right\}\cup \frac{i}{\tau }}$Res(**C**(*z*), *z*)*e*^*izt*^ for *t* ≥ 0. Sorting (17) to obtain four matrix prefactors and remainders with different frequency dependence, Φ_1_(ω) = *f*(ω)*f*(−ω), Φ_2_(ω) = *f*(ω)*f*(−ω)*H*_*d*_(ω), Φ_3_(ω) = Φ_2_(−ω), and Φ_4_(ω) = *f*(ω)*f*(−ω)*H*_*d*_(ω)*H*_*d*_(−ω), we get (18). **C**(ω) for output noise (20) is obtained by multiplying the expression for **C**(ω) for input noise with *H*^−1^_*d*_(ω)*H*^−1^_*d*_(−ω) = (1 + ω^2^τ^2^). In order to perform the back Fourier transformation one first needs to rewrite the cross spectrum in order to isolate the frequency independent term and the two terms that vanish for either *t* < *d* or *t* > *d*, as described in “Fourier back transformation,”

$$\begin{array}{l} C(\omega )=f(\omega )(1+Kw\left(\begin{array}{cc} \gamma g & -\gamma g\\ 1 & -1 \end{array}\right)H_{d}(\omega ))MDM^{T}f(-\omega )\\ \;(1+Kw\left(\begin{array}{cc} \gamma g & 1\\ -\gamma g & -1 \end{array}\right)H_{d}(-\omega ))\\ \;+f(\omega )(1+Kw\left(\begin{array}{cc} \gamma g & -\gamma g\\ 1 & -1 \end{array}\right)H_{d}(\omega ))MD\\ \;+DM^{T}f(-\omega )(1+Kw\left(\begin{array}{cc} \gamma g & 1\\ -\gamma g & -1 \end{array}\right)H_{d}(-\omega ))+D\\ \;=f(\omega )MDM^{T}f(-\omega )+f(\omega )MD+DM^{T}f(-\omega )+D, \end{array}$$

where in the last step we used $\left(\begin{array}{cc} \gamma g & -\gamma g\\ 1 & -1 \end{array}\right)M=0$, because **M** is symmetric, obtaining (21). For each of the first three terms in the last expression the right integration contour needs to be chosen as described in “Fourier back transformation” on the example of the general expression (16).

### Implementation of noisy rate models

The dynamics is propagated in time steps of duration Δ*t* (note that in other works we use *h* as a symbol for the computation step size, which here is used as the symbol for the kernel). The product of the connectivity matrix with the vector of output variables at the end of the previous step *i* − 1 is the vector **I**(*t*_*i*_) of inputs at the current step *i*. The intrinsic time scale of the system is determined by the time constant τ. For sufficiently small time steps Δ*t* « τ these inputs can be assumed to be time independent within one step. So we can use (3) or (4) and analytically convolve the kernel function *h* assuming the input to be constant over the time interval Δ*t*. This corresponds to the method of exponential integration (Rotter and Diesmann, 1999, see App. C.6) requiring only local knowledge of the connectivity matrix **w**. Note that this procedure becomes exact for Δ*t* → 0 and for finite Δ*t* is an approximation. The propagation of the initial value *r*_*j*_(*t*_*i* − 1_) until the end of the time interval takes the form *r*_*j*_(*t*_*i* − 1_) *e*^−Δ*t*/τ^ because *h*(*t*_*i*_) = *h*(*t*_*i* − 1_) *e*^−Δ*t*/τ^, so we obtain the expression *r*_*j*_(*t*_*i*_) at the end of the step as

(43) $$r_{j}(t_{i})=e^{-\Delta t/\tau }r_{j}(t_{i-1})+(1-e^{-\Delta t/\tau })I_{j}(t_{i}),$$

where *I*_*j*_ denotes the input to the neuron *j*. For output noise the output variable of neuron *j* is *y*_*j*_ = *r*_*j*_ + *x*_*j*_, with the locally generated additive noise *x*_*j*_ and hence the input is *I*_*j*_(*t*_*i*_) = (**w y**(*t*_*i*_))_*j*_. In the case of input noise the output variable is *r*_*j*_ and the additional noise is added to the input variable, *I*_*j*_(*t*_*i*_) = (**w r**(*t*_*i*_))_*j*_ + *x*_*j*_(*t*_*i*_). In both cases *x*_*j*_ is implemented as a binary noise: in each time step, *x*_*j*_ is independently and randomly chosen to be 1 or −1 with probability 0.5 multiplied with ρ/$\sqrt{\Delta t}$ to satisfy (2) for discretized time. Here the δ-function is replaced by a “rectangle” function that is constant on the interval of length Δ*t*, vanishes elsewhere, and has unit integral. The factor Δ*t*^−1^ in the expression for *x*^2^ ensures the integral to be unity. So far, the implementation assumes the synaptic delay to be zero. To implement a non-zero synaptic delay *d*, each object representing a neuron contains an array *b* of length *l*_*d*_ = *d*/Δ*t* acting as a ring buffer. The input *I*_*j*_(*t*_*i*_) used to calculate the output rate at step *i* according to (43) is then taken from position *i* mod *l*_*d*_ of this array and after that replaced by the input presently received from the network, so that the new input will be used only after one delay has passed. This sequence of buffer handling can be represented as

$$\begin{array}{l} \;I_{j}(t_{i})\leftarrow b[i\text{mod}l_{d}]\\ b[i\text{mod}l_{d}]\leftarrow \{\begin{array}{ll} {\left(wr\right)}_{j}+x_{j} & \text{for input noise}\\ {\left(wy\right)}_{j} & \text{for output noise} \end{array}. \end{array}$$

The model is implemented in Python version 2.7 (Python Software Foundation, 2008) using numpy 1.6.1 (Ascher et al., 2001) and scipy 0.9.0 (Jones et al., 2001).

### Implementation of binary neurons in a spiking simulator code

The binary neuron model is implemented in the NEST simulator, version 2.2.1 (Gewaltig and Diesmann, 2007), which allows distributed simulation on parallel machines and handles synaptic delays in the established framework for spiking neurons (Morrison et al., 2005). The name of the model is “ginzburg_neuron”. In NEST information is transmitted in form of point events, which in case of binary neurons are sent if the state of the neuron changes: one spike is sent for a down-transition and two spikes at the same time for an up-transition, so the multiplicity reflects the type of event. The logic to decode the original transitions is implemented in the function handle shown in Alg. 2. If a single spike is received, the synaptic weight *w* is subtracted from the input buffer at the position determined by the time point of the transition and the synaptic delay. In distributed simulations a single spike with multiplicity 2 sent to another machine is handled on the receiving side as two separate events with multiplicity 1 each. In order to decode this case on the receiving machine we memorize the time (*t*_last_) and origin (global id gid_last_ of the sending neuron) of the last arrived spike. If both coincide to the spike under consideration, the sending neuron has performed an up transition 0 → 1. We hence add twice the synaptic weight 2*w* to the input buffer of the target neuron, one that reflects the real change of the system state and another that compensates the subtraction of *w* after reception of the first spike of a pair. The algorithm relies on the fact that within NEST two spikes that are generated by one neuron at the same time point are delivered sequentially to the target neurons. This is assured, because neurons are updated one by one: The update propagates each neuron by a time step equal to the minimal delay *d*_min_ in the network. All spikes generated within one update step are written sequentially into the communication buffers, and finally the buffers are shipped to the other processors (Morrison et al., 2005). Hence a pair of spikes generated by one neuron within a single update step will be delivered consecutively and will not be interspersed by spikes from other neurons with the same time stamp.

The model exhibits stochastic transitions (at random points in time) between two states. The transitions are governed by probabilities ϕ(*h*). Using asynchronous update (Rumelhart et al., 1986), in each infinitesimal interval [*t, t* + δ*t*) each neuron in the network has the probability $\frac{1}{\tau }\delta t$ to be chosen for update (Hopfield, 1982). A mathematically equivalent formulation draws the time points of update independently for all neurons. For a particular neuron, the sequence of update points has exponentially distributed intervals with mean duration τ, i.e., it forms a Poisson process with rate τ^−1^. We employ the latter formulation to incorporate binary neuron models in the globally time-driven spiking simulator NEST (Gewaltig and Diesmann, 2007) and constrain the points of transition to a discrete time grid Δ*t* = 0.1 ms covering the interval *d*_min_ ≥ Δ*t*. This neuron state update is implemented by the algorithm shown in Alg. 1. Note that the field *h* is updated in steps of Δ*t* while the activity state is updated only when the current time exceeds the next potential transition point. As the last step of the activity update we draw an exponentially distributed time interval to determine the new potential transition time. The potential transition time is represented with a higher resolution (on the order of microseconds) than Δ*t* to avoid a systematic bias of the mean inter-update-interval. This update scheme is identical to the one used in (Hopfield, 1982). Note that the implementation is different from the classical asynchronous update scheme (van Vreeswijk and Sompolinsky, 1998), where in each discrete time step Δ*t* exactly one neuron is picked at random. The mean inter-update-interval (time constant τ in Alg. 1) in the latter scheme is determined by τ = Δ*tN*, with *N* the number of neurons in the network. For small time steps both schemes converge so that update times follow a Poisson process.

At each update time point the neuron state becomes 1 with the probability given by the function ϕ applied to the input at that time according to (25) and 0 with probability 1 − ϕ. The input is a function of the whole system state and is constant between spikes which indicate state changes. Each neuron therefore maintains a state variable *h* at each point in time holding the summed input and being updated by adding and subtracting the input read from the ring buffer *b* at the point readpos(t) corresponding to the current time (see Morrison et al., 2005, for the implementation of the ring buffer, i.p. Fig 6). The ring buffer enables us to implement synaptic delays. For technical reasons this implementation requires a minimal delay of a single simulation time step (Morrison and Diesmann, 2008). The gain function ϕ applied to the input *h* has the form

(44) $$\phi (h)=c_{1}h+c_{2}\frac{1}{2}(1+\tanh(c_{3}(h-\theta ))),$$

where throughout this manuscript we used *c*_1_ = 0, *c*_2_ = 1, and *c*_3_ = β, as defined in “Parameters of simulations”.

### Implementation of hawkes neurons in a spiking simulator code

Hawkes neurons (Hawkes, 1971) were introduced in the NEST simulator in version 2.2.0 (Gewaltig and Diesmann, 2007). The name of the model is “pp_psc_delta”. In the following we describe the implemented neuron model in general and mention the particular choices of parameter and correspondences to the theory presented in “Hawkes processes”. The dynamics of the quasi-membrane potential *u* is integrated exactly within a time step Δ*t* of the simulation (Rotter and Diesmann, 1999), expressing the voltage *u*(*t*_*i*_) at the end of time step *i* by the membrane potential at the end of the previous time step *u*(*t*_*i* − 1_) as

(45) $$u(t_{i})=e^{-\Delta t/\tau }u(t_{i-1})+(1-e^{-\Delta t/\tau })R_{m}I_{e}+b(t_{i}),$$

where *I*_*e*_ is a time-step wise constant input current (equal to 0 in all simulations presented in this article) and *R*_*m*_ = τ_*m*_/*C*_*m*_ is the membrane resistance. The buffer *b*(*t*_*i*_) contains the summed contributions of incoming spikes, multiplied by their respective synaptic weight, which have arrived at the neuron within the interval (*t*_*i* − 1_,*t*_*i*_]. *b* is implemented as a ring-buffer in order to handle the synaptic delay, logically similar as in “Implementation of noisy rate models,” described in detail in Morrison et al. (2005). The instantaneous spike emission rate is λ = [*c*_1_*u* + *c*_2_*e*^*c*_3_*u*^]_+_, where we use *c*_3_ = 0 in all simulations presented here. The quantities in the theory “Hawkes processes,” in particular in (35), are related to the parameters of the simulated model in the following way. The quantity *r* relates to the membrane potential *u* as *r* = *c*_1_*u* + *c*_2_ and the background rate ν agrees to *c*_2_ = ν. Hence the synaptic weight *J*_*ij*_ corresponds to the synaptic weight in the simulation multiplied by *c*_1_. For the correspondence of the Hawkes model to the OUP with output noise of variance ρ^2^ we use (38) to adjust the background rate ν in order to obtain the desired rate λ_0_ = ρ^2^ and we choose the synaptic weight *J* of the Hawkes model so that the linear coupling strength *w* of the OUP agrees to the effective linear weight given by (39). These two constraints can be fulfilled simultaneously by solving (38) and (39) by numerical iteration. The spike emission of the model is realized either with or without dead time. In this article we only used the latter. In the presence of a dead time, which is constrained to be larger than the simulation time step, at most one spike can be generated within a time step. A spike is hence emitted with the probability *p*_≥1_ = 1 − *e*^λΔ*t*^, where *e*^λΔ*t*^ is the probability of the complementary event (emitting 0 spikes), implemented by comparing a uniformly distributed random number to *p*_≥1_. The refractory period is handled as described in Morrison et al. (2005). Without refractoriness, the number of emitted spikes is drawn from a Poisson distribution with parameter λΔ*t*, implemented in the GNU Scientific Library (Galassi et al., 2006). Reproducibility of the random sequences for different numbers of processes and threads is ensured by the concept of random number generators assigned to virtual processes, as described in (Plesser et al., 2007).

### Parameters of simulations

For all simulations we used γ = 0.25 corresponding to the biologically realistic fraction of inhibitory neurons, a connectivity probability *p* = 0.1, and a simulation time step of Δ*t* = 0.1 ms. For binary neurons we measured the covariance functions with a resolution of 1 ms, for all other models the resolution is 0.1 ms. Simulation time is 10, 000 ms for linear rate and for LIF neurons, 50, 000 ms for Hawkes, and 100, 000 ms for binary neurons. The covariance is obtained for a time window of ±100 ms.

The parameters for simulations of the LIF model presented in Figure 7 and Figure 8 are *J* = 0.1 mV, τ = 20 ms, τ_*s*_ = 2 ms, τ_*r*_ = 2 ms, *V*_θ_ = 15 mV, *V*_*r*_ = 0, *g* = 6, *d* = 3 ms, *N* = 8000. The number of neurons in the corresponding networks of other models is the same. Cross covariances are measured between the summed spike trains of two disjoint populations of *N*_rec_ = 1000 neurons each. The single neuron autocovariances *a*_α_ are averaged over a subpopulation of 100 neurons. The autocovariances of the population averaged activity $\frac{1}{N_{\alpha }}a_{\alpha }+C_{\alpha \alpha }$ for population α ∈ {,} (shown in Figure 7) are constructed from the estimated single neuron population averaged autocovariances *a*_α_ and cross covariances *C*_αα_. This enables us to estimate *a*_α_ and *C*_αα_ from the activity of a small subpopulation and still assigns the correct relative weights to both contributions. The corresponding effective parameters describing the system dynamics are μ = 15 mV, σ = 10 mV, *r* = 23.6 Hz (see (40) and the following text for details).

The parameters of the Hawkes model and of the noisy rate model with output noise yielding quantitatively agreeing covariance functions are:

- For simulations of the noisy rate model with output noise presented in Figure 7 and Figure 2 the parameters are *w* = 0.0043, *g* ≈ 5.93, τ = 4.07 ms, ρ^2^ = 23.6 Hz, *d* = 3 ms (see (3), (4)). In Figure 2 also results for *d* = 1 ms and for input noise are shown. Signals are measured from *N*_rec_ = 500 neurons in each population to obtain *c*__, c__ and from the whole population to determine *c*__ and *c*__. The cross covariances *C*__ and *C*__ are estimated from two disjoint subpopulations each comprising half of the neurons of the respective population.
- For the network of Hawkes neurons presented in Figure 7 we used λ_0_ ≈ 22.54 Hz (see (38)), *J* = 0.0055 mV, *d* = 3 ms, and the same *g* and τ as for the noisy rate model. We measured the cross covariances in the same way as for the LIF model, but using the spike trains from sub-populations of *N*_rec_ = 2000 neurons. The autocovariances of the population averaged activity were estimated from the whole populations.

The network of binary neurons shown in Figure 8 uses θ = −3.89 mV, β = 0.5 mV^−1^, *J* = 0.02 mV, *d* = 3 ms (see (25), (44)), and the same *g* and τ as the noisy rate model. Covariances are measured using the signals from all neurons.

The simulation results for the network of binary neurons presented in Figure 6 uses θ = −2.5 mV, τ = 10 ms, β = 0.5 mV^−1^, *g* = 6, *J* ≈ 0.0447 mV, *N* = 2000 and the smallest possible value of synaptic delay is *d* = 0.1 ms equal to time resolution (the same set of parameters only with modified β = 1 mV^−1^ was used to create Figure 5). The cross covariances *C*__ and *C*__ are estimated from two disjoint subpopulations each comprising half of the neurons of the respective population, *c*__ is measured between two such subpopulations. For *c*__ and *c*__ we used the full populations.

The parameters required for a quantitative agreement with the rate model with input noise are *w* ≈ 0.011, ρ ≈ 2.23 $\sqrt{ms}$. We used the same parameters in Figure 3, where additionally results for *w* = 0.018 are shown. The population sizes are the same as for the binary network. The covariances are estimated in the same way as for the rate model with output noise. Note that the definition of noisy rate models has no limitation for units of ρ^2^. These can be arbitrary and are chosen differently as required by the correspondence with either spiking or binary neurons.

## References

[B1] Abeles M. (1991). Corticonics: Neural Circuits of the Cerebral Cortex. 1st Edn Cambridge: Cambridge University Press 10.1017/CBO9780511574566

[B2] Ascher D. Dubois P. F. Hinsen K. Hugunin J. Oliphant T. (2001). An Open Source Project: Numerical Python. Technical Report UCRL-MA-128569, Livermore, CA: Lawrence Livermore National Laboratory

[B3] Bi G.-Q. Poo M.-M. (1999). Distributed synaptic modification in neural networks induced by patterned stimulation. Nature 401, 792–796 10.1038/44573 10548104

[B4] Bienenstock E. (1995). A model of neocortex. Network 6, 179–224 10.1088/0954-898X/6/2/00417271176

[B5] Braitenberg V. Schüz A. (1991). Anatomy of the Cortex: Statistics and Geometry. Berlin; Heidelberg; New York: Springer-Verlag

[B6] Bronstein I. N. Semendjajew K. A. Musiol G. Mühlig H. (1999). Taschenbuch der Mathematik. 4th Edn Frankfurt am Main: Verlag Harri Deutsch

[B7] Brunel N. (2000). Dynamics of sparsely connected networks of excitatory and inhibitory spiking neurons. J. Comput. Neurosci. 8, 183–208 10.1023/A:1008925309027 10809012

[B8] Brunel N. Hakim V. (1999). Fast global oscillations in networks of integrate-and-fire neurons with low firing rates. Neural Comput. 11, 1621–1671 10.1162/089976699300016179 10490941

[B9] Buice M. A. Cowan J. D. Chow C. C. (2009). Systematic fluctuation expansion for neural network activity equations. Neural Comput. 22, 377–426 10.1162/neco.2009.02-09-960 19852585 PMC2805768

[B10] Burak Y. Lewallen S. Sompolinsky H. (2009). Stimulus-dependent correlations in threshold-crossing spiking neurons. Neural Comput. 21, 2269–2308 10.1162/neco.2009.07-08-830 19409055

[B11] Burkitt A. N. Gilson M. van Hemmen J. (2007). Spike-timing-dependent plasticity for neurons with recurrent connections. Biol. Cybern. 96, 533–546 10.1007/s00422-007-0148-2 17415586

[B12] Buzsáki G. Wang X. J. (2012). Mechanisms of gamma oscillations. Annu. Rev. Neurosci. 35, 203–225 10.1146/annurev-neuro-062111-150444 22443509 PMC4049541

[B13] Cohen M. R. Kohn A. (2011). Measuring and interpreting neuronal correlations. Nat. Rev. Neurosci. 14, 811–819 10.1038/nn.2842 21709677 PMC3586814

[B14] Corless R. M. Gonnet G. H. Hare D. E. G. Jeffrey D. J. Knuth D. E. (1996). On the lambert w function. Adv. Comput. Math. 5, 329–359 10.1007/BF02124750

[B15] Diesmann M. Gewaltig M.-O. Aertsen A. (1999). Stable propagation of synchronous spiking in cortical neural networks. Nature 402, 529–533 10.1038/990101 10591212

[B16] Fourcaud N. Brunel N. (2002). Dynamics of the firing probability of noisy integrate-and-fire neurons. Neural Comput. 14, 2057–2110 10.1162/089976602320264015 12184844

[B17] Galassi M. Davies J. Theiler J. Gough B. Jungman G. Booth M. Rossi F. (2006). GNU Scientific Library Reference Manual 2nd Edn. Bristol: Network Theory Limited

[B18] Gardiner C. W. (2004). Handbook of Stochastic Methods for Physics, Chemistry and the Natural Sciences, 3rd Edn Springer Series in Synergetics. Berlin: Springer

[B19] Gerstein G. L. Perkel D. H. (1969). Simultaneously recorded trains of action potentials: analysis and functional interpretation. Science 881, 828–830 10.1126/science.164.3881.828 5767782

[B20] Gewaltig M.-O. Diesmann M. (2007). NEST (NEural Simulation Tool). Scholarpedia 2, 1430 10.4249/scholarpedia.1430

[B21] Gilson M. Burkitt A. N. Grayden D. B. Thomas D. A. van Hemmen J. L. (2009). Emergence of network structure due to spike-timing-dependent plasticity in recurrent neuronal networks. I. Input selectivity - strengthening correlated input pathways. Biol. Cybern. 101, 81–102 10.1007/s00422-009-0319-4 19536560

[B22] Gilson M. Burkitt A. N. van Hemmen J. L. (2010). STDP in recurrent neuronal networks. Front. Comput. Neurosci. 4:23 10.3389/fncom.2010.00023 20890448 PMC2947928

[B23] Ginzburg I. Sompolinsky H. (1994). Theory of correlations in stochastic neural networks. Phys. Rev. E 50, 3171–3191 10.1103/PhysRevE.50.3171 9962363

[B24] Grytskyy D. Tetzlaff T. Diesmann M. Helias M. (2013). Invariance of covariances arises out of noise. AIP Conf. Proc. 1510, 258–262 10.1063/1.4776531

[B25] Hawkes A. (1971). Point spectra of some mutually exciting point process. J. R. Statist. Soc. Ser. B 33, 438–443

[B26] Hazewinkel M. (Ed.) (2002). Encyclopaedia of Mathematics. Dordrecht: Kluwer Academic Publishers

[B27] Helias M. Tetzlaff T. Diesmann M. (2013). Echoes in correlated neural systems. New J. Phys. 15:023002 10.1088/1367-2630/15/2/023002

[B28] Hopfield J. J. (1982). Neural networks and physical systems with emergent collective computational abilities. Proc. Natl. Acad. Sci. U.S.A. 79, 2554–2558 10.1073/pnas.79.8.2554 6953413 PMC346238

[B29] Ito J. Maldonado P. Singer W. Grün S. (2011). Saccade-related modulations of neuronal excitability support synchrony of visually elicited spikes. Cereb. Cortex 21, 2482–2497 10.1093/cercor/bhr020 21459839 PMC3183421

[B30] Jones E. Oliphant T. Peterson P. (2001). SciPy: Open Source Scientific Tools for Python. Available online at: http://www.scipy.org/

[B31] Kilavik B. E. Roux S. Ponce-Alvarez A. Confais J. Gruen S. Riehle A. (2009). Long-term modifications in motor cortical dynamics induced by intensive practice. J. Neurosci. 29, 12653–12663 10.1523/JNEUROSCI.1554-09.2009 19812340 PMC6665105

[B32] Kriener B. Tetzlaff T. Aertsen A. Diesmann M. Rotter S. (2008). Correlations and population dynamics in cortical networks. Neural Comput. 20, 2185–2226 10.1162/neco.2008.02-07-474 18439141

[B33] Markram H. Lübke J. Frotscher M. Sakmann B. (1997). Regulation of synaptic efficacy by coincidence of postsynaptic APs and EPSPs. Science 275, 213–215 10.1126/science.275.5297.213 8985014

[B34] Moreno-Bote R. Parga N. (2006). Auto- and crosscorrelograms for the spike response of leaky integrate-and-fire neurons with slow synapses. Phys. Rev. Lett. 96:028101 10.1103/PhysRevLett.96.028101 16486646

[B35] Morrison A. Diesmann M. (2008). Maintaining causality in discrete time neuronal network simulations, in Lectures in Supercomputational Neuroscience: Dynamics in Complex Brain Networks, eds beim Graben P. Zhou C. Thiel M. Kurths J. (Understanding Complex Systems, Springer), 267–278

[B36] Morrison A. Mehring C. Geisel T. Aertsen A. Diesmann M. (2005). Advancing the boundaries of high connectivity network simulation with distributed computing. Neural Comput. 17, 1776–1801 10.1162/0899766054026648 15969917

[B37] Papoulis A. Pillai S. U. (2002). Probability, Random Variables, and Stochastic Processes, 4th Edn Boston, MA: McGraw-Hill

[B38] Perkel D. H. Gerstein G. L. Moore G. P. (1967). Neuronal spike trains and stochastic point processes. II. Simultaneous spike trains. Biophys. J. 7, 419–440 10.1016/S0006-3495(67)86597-4 4292792 PMC1368069

[B39] Pernice V. Staude B. Cardanobile S. Rotter S. (2011). How structure determines correlations in neuronal networks. PLoS Comput. Biol. 7:e1002059 10.1371/journal.pcbi.1002059 21625580 PMC3098224

[B40] Pernice V. Staude B. Cardanobile S. Rotter S. (2012). Recurrent interactions in spiking networks with arbitrary topology. Phys. Rev. E 85:031916 10.1103/PhysRevE.85.031916 22587132

[B41] Plesser H. E. Eppler J. M. Morrison A. Diesmann M. Gewaltig M.-O. (2007). Efficient parallel simulation of large-scale neuronal networks on clusters of multiprocessor computers, in Euro-Par 2007: Parallel Processing, Vol. 4641 of *Lecture Notes in Computer Science*, eds Kermarrec A.-M. Bougé L. Priol T. (Berlin: Springer-Verlag), 672–681

[B42] Python Software Foundation. (2008). The Python Programming Language. Available online at: http://www.python.org

[B43] Rajan K. Abbott L. (2006). Eigenvalue spectra of random matrices for neural networks. Phys. Rev. Lett. 97:188104 10.1103/PhysRevLett.97.188104 17155583

[B44] Renart A. De La Rocha J. Bartho P. Hollender L. Parga N. Reyes A. Harris K. D. (2010). The asynchronous state in cortical cicuits. Science 327, 587–590 10.1126/science.1179850 20110507 PMC2861483

[B45] Risken H. (1996). The Fokker-Planck Equation. Verlag; Berlin; Heidelberg: Springer

[B46] Rosenbaum R. Josic K. (2011). Mechanisms that modulate the transfer of spiking correlations. Neural Comput. 23, 1261–1305 10.1162/NECO_a_00116 21299426

[B47] Rotter S. Diesmann M. (1999). Exact digital simulation of time-invariant linear systems with applications to neuronal modeling. Biol. Cybern. 81, 381–402 10.1007/s004220050570 10592015

[B48] Rumelhart D. E. McClelland J. L. the PDP Research Group (1986). Parallel Distributed Processing, Explorations in the Microstructure of Cognition: Foundations, Vol. 1 Cambridge, MA: MIT Press

[B49] Shadlen M. N. Movshon A. J. (1999). Synchrony unbound: A critical evaluation of the temporal binding hypothesis. Neuron 24, 67–77 10.1016/S0896-6273(00)80822-3 10677027

[B50] Singer W. (1999). Neuronal synchrony: a versatile code for the definition of relations? Neuron 24, 49–65 10.1016/S0896-6273(00)80821-1 10677026

[B51] Sompolinsky H. Yoon H. Kang K. Shamir M. (2001). Population coding in neuronal systems with correlated noise. Phys. Rev. E 64:51904 10.1103/PhysRevE.64.051904 11735965

[B52] Tchumatchenko T. Malyshev A. Geisel T. Volgushev M. Wolf F. (2010). Correlations and synchrony in threshold neuron models. Phys. Rev. Lett. 104, 058102 10.1103/PhysRevLett.104.058102 20366796

[B53] Tetzlaff T. Helias M. Einevoll G. Diesmann M. (2012). Decorrelation of neural-network activity by inhibitory feedback. PLoS Comput. Biol. 8:e1002596 10.1371/journal.pcbi.1002596 23133368 PMC3487539

[B54] Trousdale J. Hu Y. Shea-Brown E. Josic K. (2012). Impact of network structure and cellular response on spike time correlations. PLoS Comput. Biol. 8:e1002408 10.1371/journal.pcbi.1002408 22457608 PMC3310711

[B55] Uhlenbeck G. E. Ornstein L. S. (1930). On the theory of the brownian motion. Phys. Rev. 36, 823–841 reprinted in Wax (1954) 10.1103/PhysRev.36.823

[B56] van Vreeswijk C. Sompolinsky H. (1996). Chaos in neuronal networks with balanced excitatory and inhibitory activity. Science 274, 1724–1726 10.1126/science.274.5293.1724 8939866

[B57] van Vreeswijk C. Sompolinsky H. (1998). Chaotic balanced state in a model of cortical circuits. Neural Comput. 10, 1321–1371 10.1162/089976698300017214 9698348

[B58] von der Malsburg C. (1981). The Correlation Theory of Brain Function. Internal report 81-2, Department of Neurobiology, Max-Planck-Institute for Biophysical Chemistry, Göttingen, Germany

[B59] Wax N. (eds.). (1954). Selected Papers on Noise and Stochastic Processes. New York, NY: Dover Publications

[B60] Zohary E. Shadlen M. N. Newsome W. T. (1994). Correlated neuronal discharge rate and its implications for psychophysical performance. Nature 370, 140–143 10.1038/370140a0 8022482

